# The hazards of metal exposure mediated by crops to human health from perspective of environmental health and control strategies

**DOI:** 10.1016/j.isci.2026.116147

**Published:** 2026-06-08

**Authors:** Huanhuan Yang, Fulan Zhang, Jiacun Zhou, Jiyao Zhu, Dayong Cui, Xu Zhang, Zhibin Zhang

**Affiliations:** 1 School of Life Sciences, Qilu Normal University, Jinan 250200, China; 2 The College of Life and Geographic Sciences, Kashi University, Kashi 844000, China; 3 School of Municipal and Environmental Engineering, Shandong Jianzhu University, Jinan 250101, China

**Keywords:** health technology, hazard identification, applied sciences

## Abstract

Heavy metal pollution, driven by rapid industrialization, urbanization, and agricultural intensification, poses a significant threat to ecosystem security and human health. This review examines soil-to-crop-to-human transfer pathways of cadmium (Cd), lead (Pb), mercury (Hg), arsenic (As), and chromium (Cr), covering their environmental behavior, crop accumulation, and toxicological impacts (neurotoxicity, nephrotoxicity, and carcinogenicity). We discuss plant molecular stress responses (oxidative damage, enzyme disruption, and genetic alteration) and synthesize integrated control strategies including source control, soil remediation, agronomic management, and medical interventions. Emerging technologies (nanomaterials, phytoremediation, AI monitoring) are highlighted. We emphasize the importance of a holistic “source-process-risk-population” approach to effectively reduce heavy metal exposure via crops. This work aims to support environmental improvement, sustainable agriculture, and public health protection, contributing to national and global goals for food safety and human well-being.

## Introduction

**Figure 1:**
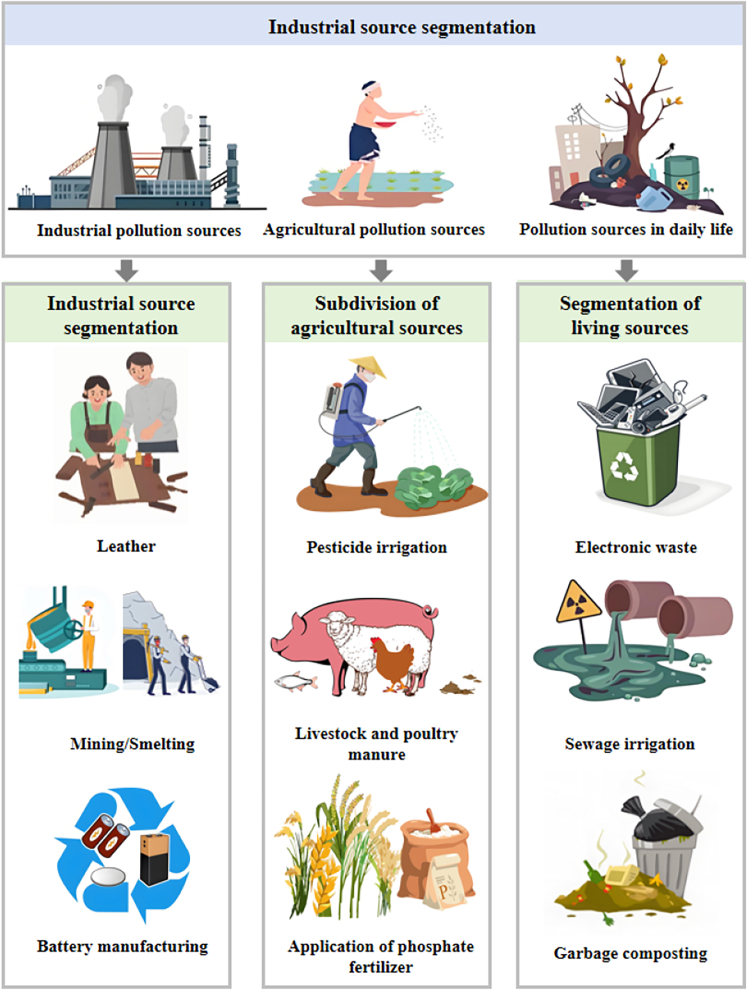
Status and main sources of heavy metal pollution (Image source, drawn by the author).

With the acceleration of global industrialization and agriculture, heavy metal pollution in arable land has become a significant environmental issue threatening ecosystems and food security.^1^ As shown in Figure 1, heavy metals are continuously introduced into environmental media through mining, smelting emissions, transportation, coal combustion, fertilizer and pesticide application, wastewater irrigation, and electronic waste dismantling.^2^ Due to their persistence, bioaccumulation, and long-term toxicity, they persist in the soil-water-atmosphere system.^3^ It is estimated that approximately 14%–17% of the world’s arable land is contaminated by at least one heavy metal, covering an area exceeding 240 million hectares, potentially affecting 900 million to 1.4 billion people.^4^

Historical cases demonstrate that the hazards of heavy metal pollution are long-term, latent, and cumulative.^5^ Japan’s “Minamata disease” revealed the severe neurotoxicity caused by methylmercury transfer through the food chain^6^; “Itai-itai disease” showed the serious consequences of cadmium (Cd) accumulation in rice, leading to osteomalacia and renal damage. These events not only revealed chronic health risks but also spurred international control actions, such as the implementation of the Minamata Convention.

Notably, the coupling effects of heavy metal pollution with climate change, land use patterns, and agricultural production models are intensifying. For instance, alternate wet and dry irrigation regimes, acidic soil environments, and changes in organic matter content significantly influence the bioavailability and crop uptake of metals like Cd and arsenic (As).^7^ Therefore, systematically researching the entire chain—from pollution sources to crop exposure to health effects—is crucial.

Despite substantial progress in understanding individual aspects of heavy metal pollution, several critical scientific challenges remain unresolved along the soil-crop-human continuum:

Coupling mechanisms between metal speciation and crop uptake is one primary challenge: the bioavailability of heavy metals is governed by complex interactions among soil physicochemical properties (pH, redox potential, and organic matter), rhizosphere processes, and metal speciation. However, predictive models that quantitatively link these factors to crop accumulation remain underdeveloped, particularly under dynamic field conditions influenced by climate change and agronomic practices.

Combined pollution and joint toxicity is another relevant factor: agricultural soils are typically contaminated with multiple metals; yet, most studies focus on single-metal effects. The antagonistic or synergistic interactions among co-occurring metals, and their combined effects on crop physiology and human health, remain poorly characterized.

Trade-offs in mitigation strategies are another challenging area: current prevention measures—including soil remediation, agronomic management, and breeding of low-accumulation varieties—often involve trade-offs between efficacy, cost, environmental impact, and food security. A systematic framework for evaluating and integrating these approaches is lacking.

Addressing these challenges requires a holistic perspective that integrates environmental geochemistry, plant physiology, toxicology, and public health. This review is structured around the pathway of “pollution sources-crop-mediated exposure-health effects-integrated control.” It aims to comprehensively summarize the pollution status, migration mechanisms, and toxicological impacts of major heavy metals. By integrating the latest scientific progress, it discusses targeted strategies for pollution interception and health risk intervention, providing theoretical support for environmental management, agricultural transformation, and the advancement of global sustainable development goals.

## Main types of soil heavy metal pollutants and their hazards

With the development of industrialization, urbanization, and agricultural intensification, the problem of soil heavy metal pollution is becoming increasingly severe worldwide.^8^ Common polluting heavy metals in soil include As, Cd, chromium (Cr), lead (Pb), mercury (Hg), copper (Cu), nickel (Ni), zinc (Zn), and cobalt (Co). These elements are persistent, bioaccumulative, and toxic. Once entering the ecosystem, they readily accumulate in crops and the food chain, posing long-term threats to ecological security and human health. The World Health Organization (WHO) estimates that Pb exposure alone causes over 1.5 million deaths annually, with particularly significant impacts on children’s intellectual development. Furthermore, the disease burden from heavy metal exposure is enormous (exceeding 33 million DALYs). Recent advances in heavy metal toxicology have provided deeper insights into the molecular mechanisms underlying metal-induced toxicity. A comprehensive review by Jomova et al. systematically updates the understanding of how Cd, Pb, Hg, As, and Cr disrupt cellular redox homeostasis, interfere with mitochondrial function, and trigger programmed cell death pathways. Notably, the authors highlight emerging evidence that heavy metals induce ferroptosis—an iron-dependent form of regulated cell death—as a novel mechanism contributing to nephrotoxicity and neurotoxicity, expanding the classical paradigm of oxidative stress-mediated damage.^3^

### Cadmium

Cadmium primarily originates from industrial emissions such as electroplating, batteries, pigments, plastic stabilizers, and phosphate fertilizer production, as well as from sewage irrigation and atmospheric deposition (e.g., vehicle exhaust).^9^ It is one of the most toxic heavy metals. Cadmium is relatively mobile in soil and is easily absorbed by crops, especially rice and vegetables.^10^ Excessive Cd in soil leads to high concentrations accumulating in the edible parts of crops (e.g., “cadmium rice”). After entering the human body, it mainly accumulates in the kidneys and bones. Long-term intake can cause renal dysfunction, osteoporosis, bone deformation (i.e., “Itai-itai disease”), and may also affect the reproductive and nervous systems.^11^

Rice is a typical crop for Cd absorption, with grains exhibiting a prominent accumulation capacity.^12^ The “cadmium rice” phenomenon occurs frequently; for example, Cd levels in rice grains from some polluted areas in Hunan often exceed standards. Additionally, leafy vegetables like spinach and pak choi have a strong capacity to absorb Cd.^13^ When grown in Cd-contaminated soil, Cd levels in their leaves easily exceed safety standards. Root and tuber crops such as potatoes and carrots also absorb Cd from soil and accumulate it in their tubers or fleshy roots.

Recent research indicates that the global background Cd concentration in arable soils generally ranges from 0.1 to 0.5 mg/kg, but soils in areas severely affected by industrial activities, such as mining areas, can have Cd concentrations as high as 20 mg/kg. Long-term Cd intake can Pb to chronic kidney damage. The International Agency for Research on Cancer (IARC) classifies Cd as a group 1 carcinogen. Japan’s “Itai-itai disease” in Toyama prefecture revealed the cumulative nature of Cd in the food chain, becoming a classic case of chronic metal poisoning. The environmental behavior and health risks of Cd are summarized in Table 1.

**Table 1:** Overview of cadmium environmental behavior and health risks

Indicator	Specific content
**Main sources**	Industrial smelting, mine wastewater, phosphate fertilizer application, sewage irrigation, municipal waste incineration fly ash, traffic emissions, etc.
**Soil concentration range**	Background, 0.1–0.5 mg/kg; polluted areas, up to 20 mg/kg
**Typical accumulating crops**	Rice, spinach, rapeseed, pak choi, potato, carrot, etc.
**Human target organs**	Kidneys, bones, liver, lungs
**Main health hazards**	Renal damage, osteoporosis, osteomalacia, increased cancer risk; endocrine disruption
**Classic case**	Japan’s “Itai-itai disease”, residents suffered skeletal deformities due to cadmium-contaminated rice intake.
**Environmental and food limits**	China, farmland soil Cd limit 0.3–0.6 mg/kg; rice Cd limit 0.2 mg/kg (GB2762-2022) EU, wheat Cd limit 0.2 mg/kg (EC 1881/2006); soil Cd limit 1–3 mg/kg (depending on pH, EU Directive 86/278/EEC) Japan, rice Cd limit 0.4 mg/kg (Food Sanitation Act) CAC, rice Cd limit 0.4 mg/kg (CXS 193–1995)

Note, the table and all related contents were compiled by the author.

### Lead

Lead mainly comes from vehicle exhaust, battery manufacturing, metal smelting, paints and coatings, and waste incineration.^14^ Lead is stable in soil, not easily degraded, and can be absorbed by crop roots (e.g., wheat, vegetables, etc.), with leafy vegetables having a stronger absorption capacity. Lead poses significant harm to the human nervous system (especially in children), leading to intellectual developmental delay and cognitive decline, while also damaging the hematopoietic system and kidneys.^15^ Studies show that children chronically exposed to lead-polluted environments have significantly lower intelligence quotient (IQ) levels than normal children, with difficulties in concentration and greatly impaired learning ability.

The main vegetable varieties absorbing Pb are leafy vegetables like spinach, lettuce, and Indian lettuce, whose leaves have a significant Pb accumulation capacity. When grown in lead-polluted areas, Pb content in the leaves is often high. Additionally, celery stems and leaves can also absorb considerable lead, related to their growth characteristics and Pb absorption mechanisms.^16^

Global surveys indicate that soil Pb concentrations are commonly well above safe limits in historical industrial areas. In Wales, UK, over 1,300 abandoned mine sites release large amounts of Pb into the environment annually, keeping blood Pb levels in surrounding residents at high risk long-term. The WHO reports that in 2021, Pb exposure caused over 1.5 million deaths, and approximately 800 million children had blood Pb levels ≥5 μg/dL. Lead poisoning remains a major environmental risk factor for cognitive impairment and behavioral problems in children.^17^ Table 2 provides an overview of lead’s environmental behavior, sources, and associated health risks.

**Table 2:** Overview of lead environmental behavior and health risks

Indicator	Specific content
**Main sources**	Lead-acid battery industry, traffic exhaust, smelting wastewater, paints and coatings, old housing lead-containing materials, industrial dust
**Soil concentration range**	Background, 20–40 mg/kg; polluted areas, can exceed 1,000 mg/kg
**Typical accumulating crops**	Spinach, lettuce, celery, root vegetables
**Human target organs**	Nervous system, kidneys, bones, blood
**Main health hazards**	Intellectual decline in children, cognitive impairment, anemia, hypertension, renal damage
**Classic case**	The lead pollution crisis in Flint drinking water, USA In some mining areas of China, children’s blood lead levels have long exceeded the standard
**Environmental and food limits**	China, soil Pb limit ≤80 mg/kg; drinking water Pb limit 0.01 mg/L (GB5749-2022) EU, drinking water Pb limit 0.005 mg/L (EU 2020/2184); vegetable Pb limit 0.3 mg/kg (EC 1881/2006) Japan, drinking water Pb limit 0.01 mg/L (Water Supply Act) CAC, drinking water Pb limit 0.01 mg/L (WHO Guidelines for Drinking-water Quality)

Note, the table and all related contents were compiled by the author.

### Mercury

Mercury primarily originates from chemical industry, smelting, instrument manufacturing, Hg-containing pesticides (e.g., methylmercury), and coal combustion emissions. Methylmercury is more biologically toxic. Mercury in soil can be converted to the more toxic methylmercury, entering the food chain through crops like rice.^18^ Methylmercury has strong neurotoxicity, damaging the central nervous system, manifesting as ataxia, visual and auditory impairment, etc.^19^

Rice has a strong capacity to absorb and accumulate Hg (especially methylmercury).^20^ In Hg-polluted soil, Hg content in rice grains may exceed standards. Secondly, solanaceous fruits like tomatoes and eggplants also absorb certain amounts of Hg, accumulating it in their fruits. The Minamata Convention came into force in 2017, aiming to reduce global Hg emissions and use. Research shows that Hg concentration in paddy soil in polluted areas can exceed 10 mg/kg. According to China’s “Soil Environmental Quality – Agricultural Land Soil Pollution Risk Control Standards (Trial)” (GB 15618-2022), the risk screening value for Hg in paddy soil is 0.5 mg/kg (pH ≤ 6.5). Mercury poses particularly high risks to pregnant women and fetuses; fetal exposure can lead to severe neurodevelopmental disorders.^21^ Key characteristics of Hg pollution and its health impacts are summarized in Table S1.

### Arsenic

Although As is a metalloid, it is often included in heavy metal pollution studies. Its main sources include mining, smelting, pesticides (e.g., lead arsenate), coal combustion, and phosphate fertilizer production. Arsenic is easily absorbed by plants in soil, especially root vegetables and rice. Soil As pollution can Pb to excessive As content in crops (e.g., root vegetables).^22^ Short-term high intake can cause acute poisoning (vomiting, diarrhea, shock, etc.), while long-term low-dose intake increases cancer risk (e.g., skin, lung, bladder cancer, etc.) and damages the cardiovascular and nervous systems. *Pteris vittata* (brake fern), though not an edible crop, is a hyperaccumulator of As, absorbing large amounts from soil.^23^ Among edible crops, root and tuber crops like sweet potatoes and potatoes have a strong As absorption capacity. Additionally, rice absorbs As from soil, especially in acidic soil conditions where uptake is more pronounced.^24^

Arsenic often exists in the environment as inorganic As, with trivalent As (As^3+^) being the most toxic, readily binding to protein thiol groups and interfering with metabolic enzyme activity.^25^ Long-term consumption of water with excessive As leads to keratinization of the skin, dark spots and various types of cancer.^26^ In China, soil As concentrations around some mines and smelters are significantly higher than background levels, with As content in some rice exceeding food safety limits (0.2 mg/kg). In recent years, As pollution has also become an important risk factor for farmland ecological security in the Yellow river basin and the middle-lower Yangtze river region. The environmental behavior, crop accumulation, and health hazards of As are summarized in Table S2.

### Chromium

Chromium primarily comes from industrial wastewater and slag from electroplating, leather tanning, metal smelting, and pigment manufacturing.^26^ Chromium exists in multiple valence states, with its toxicity closely related to its state; hexavalent Cr (Cr (VI)) is far more toxic than trivalent Cr (Cr (III)) and is more easily absorbed by plants.^27^ Cr (VI) is strongly oxidizing and carcinogenic. After entering the human body, it can damage the digestive and respiratory tracts. Long-term exposure increases the risk of lung and skin cancer and may Pb to liver and kidney dysfunction.^28^ The Cr absorption capacity of wheat cannot be ignored; in Cr-contaminated soil, Cr content in wheat grains and stems increases.^29^ Simultaneously, some leafy vegetables like mustard greens and amaranth can also absorb significant amounts of Cr.

Hexavalent Cr (Cr^6+^) is classified as a group 1 carcinogen by IARC. Studies show that soil Cr concentrations near tanneries, electroplating plants, and dye industries can exceed 400 mg/kg, far above risk screening values. Chromium has strong oxidizing properties, can cross cell membranes, and induce DNA damage and cell mutation.^30^ Chromium slag dumping has caused soil and groundwater pollution incidents in many places domestically and internationally. For example, soil Cr concentration at a Cr slag dump site in Guizhou province, China, exceeded 1,500 mg/kg, posing a serious threat to the health of surrounding residents. Regarding crops, research indicates that vegetables like spinach and carrots have a certain capacity to accumulate Cr, especially under Cr (VI) pollution conditions where accumulation is more significant.^31^ Table S3 provides a comprehensive overview of Cr’s environmental behavior and associated health risks.

### Other heavy metals

Cu and Zn mainly come from mining, smelting, pesticides (e.g., copper sulfate), and livestock manure (Cu/Zn additives in feed).^32^ Cu and Zn are essential trace elements for crop growth, but excessive accumulation inhibits growth (e.g., blocked root development, leaf chlorosis, etc.).^33^ In humans, excess Cu damages the liver and nervous system, while excess Zn may interfere with the absorption of other trace elements (e.g., iron and Cu), causing metabolic disorders.^34^

Nickel (Ni) primarily originates from steel smelting, electroplating, battery manufacturing, and fossil fuel combustion. Nickel is easily absorbed by plants in soil. Long-term intake increases the risk of lung and nasopharyngeal cancer and may cause skin allergies.^35^ Excess Co may damage heart and thyroid function and affect the hematopoietic system.

Copper and Zn are essential nutrients for humans within appropriate ranges, participating in various metabolic processes.^34^ However, excessive accumulation can cause liver, kidney, and immune system damage. Nickel and Co mainly come from smelting, battery manufacturing, and hard alloy processing industries.^36^ Nickel is classified as a group 1 carcinogen by IARC, causing nasopharyngeal cancer, lung cancer, and dermatitis; excess Co can Pb to myocardial damage and thyroid abnormalities. In recent years, the rapid development of the battery industry has significantly increased soil Co content in some industrial areas, exceeding background levels by 3–5 times. These heavy metals are persistent, easily accumulated in soil, and can be biomagnified through the food chain. Long-term low-concentration exposure may also cause irreversible harm to ecosystems and human health. Therefore, controlling heavy metal pollution sources and remediating contaminated soil are important tasks for environmental protection. The environmental behavior, sources, crop accumulation patterns, and health hazards of Cu, Zn, Ni, and Co are summarized in Table S4.

## Main sources of soil heavy metal pollution

### Industrial pollution sources

Wastewater and slag generated during metal mining and smelting contain large amounts of heavy metals (e.g., lead, Cd, Zn, etc.), which enter the soil through leakage or accumulation.^37^ Taking Pb-Zn mining as an example, processes like ore crushing and beneficiation produce large amounts of tailings containing high concentrations of Pb, Zn, and other heavy metals. Long-term stockpiling of these tailings around mining areas allows heavy metals to continuously permeate the soil through rainwater leaching, causing severe pollution in surrounding soils.^38^ Studies show that Pb content in soil around a certain lead-Zn mine can reach thousands of mg/kg, far exceeding soil environmental quality standards.

Industrial sources are among the primary pathways for heavy metal accumulation in soil and crops, including tailings, slag, and wastewater discharge from mining, smelting, electroplating, leather, dyeing, and chemical industries.^39^ Research indicates that soil Pb and Zn concentrations around smelting areas can reach thousands to tens of thousands mg/kg. For example, near a Pb-Zn smelter, surface soil Pb reached 3,975–26,200 mg/kg, Zn 3,358–21,867 mg/kg, far exceeding background values for farmland soil. Heavy metals enter soil via dust settlement or acidic wastewater. Their activity increases in acidic or reducing environments, facilitating plant uptake via root ion channels or complexed forms. Cd mainly enters through IRT1/ZIP transporters,^40^ As (III) through Lsi1/Lsi2 aquaporins,^41^ and Pb can enter roots via calcium channels or surface binding. E-waste dismantling sites are also high-risk industrial sources. In places like Guiyu, Guangdong, soil and sediment levels of Pb, Cu, Cd, etc., are elevated, with heavy metal content in rice and leafy vegetables significantly exceeding the standard, posing potential health risks to residents. Studies show that Pb-Zn smelting and e-waste dismantling areas are significant hotspots for global farmland heavy metal pollution. These pollutants distribute within plants via xylem and phloem, with leaf and grain content influenced by soil pH,^42^ organic matter, Fe/Mn oxide content, etc. This indicates that industrial activities are the core driving factor for the formation of high-concentration pollution zones. Integrated management measures combining source control, isolation/interception, and agronomic regulation should be implemented in polluted areas. The input characteristics and risk management measures for industrial pollution sources are summarized in Table 3.

**Table 3:** Heavy metal input characteristics and risk management measures for industrial pollution sources

industrial pollution input pathway	Typical metals	Risk parameters/range	High-risk crops	Risk characteristics	Management suggestions
**Pb-Zn smelting and slag areas**	Pb, Zn, Cd	Pb, 3,975–26,200; Zn, 3,358–21,867	Leafy > root; rice	Deposition of smoke and dust particles and acidic leachate; edible parts Pb/Cd exceeding the standard	Source emission control, slag cover/isolation; pH adjustment, low-accumulation crops
**Electroplating, leather, dyeing**	Cr(VI), Ni	Cr, 400–1,500; Ni, 100–500	Spinach, carrot	High toxicity of Cr(VI), strong oxidizer; Ni easily absorbed via complexes	Sludge/wastewater pretreatment; raised bed planting; Fe oxides/organic matter immobilization
**Gold mining, Hg processing areas**	Hg, As	Hg-polluted area, >10; As, 50–300	Rice, vegetables	Methylmercury biomagnification; high Hg risk in rice grains	Zero wastewater discharge, wetland barriers; switch to low-accumulation crops; ban high-risk produce
**E-waste dismantling areas**	Pb, Cu, Cd, Hg, Sb, Ni	Pb > 500, Cu > 400, Cd > 5 (multiple standards exceeded)	Rice, Garden crops	Acid washing, incineration cause soil/air pollution; significant enrichment in garden/rice crops	Curb informal sites; soil cleanup/replacement; enclosure isolation; urban-rural planning risk assessment

Note, the table and all related contents were compiled by the author.

### Agricultural pollution sources

Phosphate fertilizers often contain heavy metals like Cd and lead; long-term application leads to soil accumulation.^43^ Unreasonable use of pesticides containing As, Cu, etc. (e.g., lead arsenate, copper sulfate, etc.) is also an important source. Research finds that long-term, heavy application of a certain brand of phosphate fertilizer gradually increases soil Cd content, averaging an annual increase of 0.01–0.03 mg/kg. The use of As pesticides like lead arsenate not only directly pollutes soil but also leaves residues in crops, harming human health.^44^ Using untreated industrial or domestic wastewater for irrigation introduces heavy metals into soil. In some water-scarce areas, farmers use untreated wastewater for irrigation, causing a sharp rise in soil heavy metal content. For example, long-term wastewater irrigation in rural areas around a city led to Cd, Hg, and Pb levels several to tens of times higher than background values, with heavy metal content in grown crops severely exceeding the standard.^45^ Cu, Zn, and other trace elements added to feed enter soil through manure excretion, leading to excessive accumulation. With the scaling of livestock farming, high doses of Cu, Zn, etc., are often added to feed to promote growth.^46^ Most of these elements are not fully absorbed by livestock and are excreted into soil. Studies show that soil Cu content around large livestock farms can be as high as 1,000 mg/kg, far exceeding normal ranges, negatively impacting soil ecosystems and crop growth.

Agricultural inputs are important chronic sources of heavy metal accumulation in farmland soil. Phosphate fertilizers, due to natural Cd impurities, cause soil Cd accumulation with long-term use.^47^ EU Regulation (EU 2019/1009) has set Cd limits, gradually tightening from 60 mg/kg P_2_O_5_ to 20 mg/kg P_2_O_5_ to reduce risk. Municipal sludge and biosolids used as organic fertilizers can input Cu, Zn, Pb, Cd, etc. The Sludge Directive 86/278/EEC requires strict testing of soil and sludge metal content (e.g., Cd 20–40 mg/kg dry solids, Pb 750–1,200 mg/kg) and sets application limits. Global surveys indicate 29.3 million hectares of downstream irrigated farmland use low-level treated or untreated urban wastewater, directly affecting 88.5 million people, especially in Asia and Africa. Livestock manure, due to high usage of Cu/Zn additives in feed, is also a significant source of metal input to farmland.^48^ Cu/Zn content in vegetable plots and grain fields with years of manure application is significantly higher than controls. Furthermore, legacy As pesticides in historical orchards are a hallmark source of co-existing Pb/As pollution, with long-term impacts on land redevelopment and edible crop safety. These facts show that agricultural inputs are important diffuse sources of heavy metal pollution. Management strategies should combine source limits, regular testing of fertilizer and sludge heavy metals, pH adjustment, and breeding low-accumulation varieties to reduce agricultural product risks. Table S5 summarizes the heavy metal input characteristics and risk management measures for agricultural pollution sources.

### Domestic pollution sources

Although the overall contribution of domestic sources to heavy metal pollution is less than industrial and agricultural sources, it exhibits characteristics of dispersion, locality, and high sensitivity,^49^ particularly impacting small-scale environments like urban peripheral gardens, backyard plots, and subsistence rice paddies. In low-income and some middle-income countries, the direct use of inadequately treated domestic sewage and greywater for suburban farmland irrigation has become a common pathway for dietary heavy metal exposure,^50^ posing both pathogenic microbial risks and introducing metals like Cd, Pb, Cu, and Zn. Global spatial analysis shows approximately 29.3 million hectares of farmland rely on low-treatment-level or untreated wastewater for irrigation, affecting a population of about 88.5 million in the relevant watersheds. Long-term accumulation significantly elevates heavy metal levels in farmland soil and garden plots.

Urban municipal solid waste compost and homemade organic fertilizers, if mixed with waste batteries, metal paints, and electronic components, can significantly increase Pb, Cd content in compost.^50^ Long-term use leads to increased soil metal pools, with Cd concentrations in some leafy vegetables approaching or exceeding the 0.2 mg/kg food safety limit. Informal e-waste dismantling and metal recycling sites are often concentrated on urban fringes. Soil and ditch sediments in these areas commonly have exceeding the standard levels of Pb, Cu, Cd, and Hg.^51^ Blood Pb and urinary Cd levels in residents near family gardens and irrigated paddies are significantly higher than control areas. Multiple studies in Guiyu, Guangdong, indicate that long-term open-air burning and acid washing operations have caused severe pollution in regional farmland and the food chain.

Additionally, historical pesticide-contaminated land is also an important component of domestic source risk.^52^ Guidelines in the US, China, and other countries require soil testing and remediation before developing old orchards or land with a history of lead arsenate pesticide use into residential areas or gardens, as such land often poses combined pollution risks from Pb and As, with children being the most sensitive exposure group.

Overall, domestic heavy metal pollution is characterized by point-source concentration, high community sensitivity, and complex exposure pathways, including intake from homegrown produce, inhalation, and hand-to-mouth contact. Governance strategies require multi-level coordination^52^: ① promote hazardous waste sorting and recycling, raise public awareness of compost and organic fertilizer safety; ② strengthen urban wastewater treatment and gardening safety management; ③ implement standardized centralized management for e-waste dismantling and metal recycling sites; and ④ conduct surveys and safe use planning for high-risk historical land. Combining community governance and public education can effectively reduce heavy metal exposure risks from self-grown crops. Table S6 presents the risk characteristics and management suggestions for domestic pollution sources.

### Recent global case studies and statistical evidence

The magnitude of heavy metal contamination in agricultural systems has been underscored by recent large-scale assessments. A landmark study published in *Science* (2025) compiled data from nearly 800,000 soil samples worldwide and estimated that 14%–17% of global cropland—approximately 240 million hectares—is contaminated by at least one toxic metal (As, Cd, Pb, Cr, Ni, or Co), potentially affecting 900 million to 1.4 billion people.^4^ Notably, the study identified a previously unrecognized high-risk corridor spanning low-latitude Eurasia, where natural geological factors and long-term industrial activity have converged to create elevated metal concentrations.^53^

Regional case studies further illustrate the diverse pathways and impacts of contamination. In northwestern China, a field study near smelting facilities in Jinchang City (the “Nickel Capital”) found that mean soil concentrations of nickel (143.66 mg/kg), Cu (130.00 mg/kg), and Co (24.04 mg/kg) all exceeded regional background values, with HI calculations indicating elevated non-carcinogenic health risks for children consuming locally grown wheat and corn.^54^ A separate investigation in Nigeria revealed a previously underestimated pathway: Hg from artisanal gold mining contaminates crops primarily through atmospheric uptake rather than root absorption; crops grown 500 m from artisanal and small-scale gold mining sites had Hg concentrations 10–50 times higher than those grown 8 km away, demonstrating that airborne transport can cause significant contamination even without direct soil contact. Together, these global and regional findings underscore the urgent need for coordinated monitoring, remediation, and policy interventions to safeguard food systems and public health.

## Pathways of heavy metal entry into crops and accumulation sites

### Pathways of heavy metal entry into crops

Roots are the primary pathway for heavy metal entry into crops. Heavy metals in soil in ionic (e.g., Cd^2+^, Pb^2+^) or complexed forms are actively or passively absorbed by crop roots.^55^ Absorption efficiency is regulated by various environmental factors like soil pH, organic matter content, and heavy metal chemical speciation.^56^^,^^57^ For example, in acidic soil, heavy metal bioavailability is usually higher, making them more easily absorbed by crops. Studies show that rice root Cd absorption efficiency in acidic soil is several times higher than in neutral or alkaline soil.^58^ This is because under acidic conditions, higher hydrogen ion concentration competes with heavy metal ions for adsorption sites, weakening soil particle adsorption capacity for heavy metal ions, thereby increasing their activity and bioavailability, promoting migration to and absorption by roots.

Soil pH, redox potential (Eh), organic matter, and Fe/Mn oxides significantly influence heavy metal availability. Acidic conditions enhance the activity of Cd, Pb, Ni, etc. Reducing conditions promote the reduction of As(V) to the more absorbable As(III), enhancing As accumulation in rice grains. Rhizosphere-secreted organic acids (e.g., citric, oxalic) and amino acids (histidine) can complex Zn, Ni, etc., increasing mobility^59^; flavonoids regulate rhizosphere microbial communities and influence metal immobilization. Different metals enter plants via different transmembrane channels (see Section 4.3 for detailed transporter mechanisms). Rhizosphere iron plaques and mycorrhizal fungi play important roles in metal immobilization and barrier functions.^60^

Cation exchange capacity (CEC) is another critical determinant; soils with high CEC (e.g., clay-rich or high-organic matter soils) retain more cationic metals, reducing bioavailability, while sandy soils with low CEC are more vulnerable to metal leaching and plant uptake. Soil mineral composition, particularly the presence of clay minerals (e.g., montmorillonite) and carbonates, also contributes to metal fixation through interlayer trapping or precipitation.

While soil physicochemical factors broadly influence metal bioavailability, the geochemical behaviors of individual metals differ markedly. Cd is highly pH-sensitive and readily translocates to grains; Pb forms insoluble precipitates and is largely retained in roots. As behaves as an oxyanion—bioavailability increases under reducing conditions as As (V) reduces to As (III), which is taken up via silicon transporters, creating a trade-off in flooded paddies where Cd decreases but As increases. Cr exhibits valence-dependent behavior: Cr (VI) is highly mobile and toxic, while Cr (III) is relatively insoluble. Cu and Zn are essential micronutrients whose bioavailability is dominated by organic complexation. These differences necessitate metal-specific management strategies, such as phosphate amendments for Pb immobilization, redox manipulation for Cr, and integrated water-amendment approaches for Cd-As co-contaminated soils. Key environmental factors influencing rhizosphere heavy metal uptake are summarized in Table S7.

### Accumulation sites of heavy metals in crops

#### Roots

Most heavy metals (e.g., Pb, Cr, etc.) accumulate highest in roots, as they can be adsorbed or fixed by root cell walls,^61^ with weak translocation to shoots. Studies find that Pb accumulation in corn roots is several times that in aboveground parts.^62^ Lead is mainly fixed in pectin and cellulose components of root cell walls, limiting its transport upwards.^63^ These components contain numerous functional groups (carboxyl, hydroxyl) that can complex or exchange ions with Pb ions, adsorbing and fixing them in the cell wall.

#### Leaves

Leafy vegetables (e.g., spinach, lettuce) have a strong capacity to accumulate Cd, Pb, As, etc., in their leaves, making them the primary accumulation site.^64^ For example, Cd content in spinach leaves can account for 70%–80% of the total plant Cd. This is because leafy crops have short growth cycles, and heavy metals absorbed by roots accumulate mainly in leaves before being fully translocated elsewhere. Simultaneously, the physiological structure of leaves gives them a strong capacity to absorb heavy metals from atmospheric deposition, further increasing leaf heavy metal content.^65^

#### Grains/fruits

Grains of cereal crops like rice and wheat easily accumulate Cd, Hg (e.g., rice grain Cd can exceed safety standards).^66^ Heavy metal accumulation in fruits is relatively low, but long-term pollution can still Pb to exceeding the standard. In severely Cd-polluted paddies, rice grain Cd content often exceeds national food safety standards by several times.^67^ This is because during the grain filling stage, Cd absorbed by roots is translocated and accumulated in grains via xylem and phloem.^68^ Although fruits have a relatively weak accumulation capacity, long-term exposure to polluted environments gradually increases heavy metal content. For example, apples grown in lead-polluted areas have slightly higher Pb content than those from normal areas.

#### Stems

Heavy metal accumulation in stems is typically intermediate between roots and leaves/grains, primarily transported via xylem or phloem. In soybeans, for instance, Cd content in stems is higher than in grains but lower than in roots and leaves.^69^ During soybean growth, part of the Cd absorbed by roots is transported upwards to leaves and stems via xylem, while another part is redistributed among organs via phloem. Thus, stems become an important site for heavy metal transport and accumulation, but due to their physiological functions, accumulation is generally lower than in roots and leaves.^70^

#### Comparative accumulation patterns across crop categories and dietary risk implications

Different crop categories exhibit distinct heavy metal accumulation patterns, which directly influence dietary exposure risks. Cereal crops (rice, wheat, corn, etc.) primarily accumulate metals in grains, with rice showing particularly strong affinity for Cd and As; the dietary contribution of cereals often exceeds 50% of total Cd intake in rice-consuming populations.^71^ Leafy vegetables (spinach, lettuce, pak choi, etc.) have the highest accumulation capacity among vegetables, especially for Pb and Cd, due to their large leaf surface area and active transpiration, posing elevated risks when grown near pollution sources. Root and tuber crops (carrot, potato, radish, etc.) tend to concentrate metals in periderm tissues, with peeling effectively reducing exposure by 30%–50%. Fruit crops (tomato, eggplant, apple, etc.) generally exhibit the lowest accumulation, as metals are partially retained in leaves and stems rather than translocated to fruits.^72^ These category-specific patterns underscore the need for differentiated risk management strategies, such as selecting low-accumulation cereal varieties, siting leafy vegetable production away from industrial areas, promoting peeling of root crops, and prioritizing fruit crops for safer consumption in moderately contaminated regions.

### Accumulation differences and dietary risks

After crossing the cell membrane into plant cells, heavy metals are transported within the plant via xylem and phloem.^73^ The migration ability of different metals within plants varies significantly; some are fixed in roots, others translocate to leaves, stems, and grains. Plants utilize various transmembrane proteins for metal transport and distribution. Cd is transported from roots to shoots via heavy metal ATPase (HMA) 2/3 and natural resistance-associated macrophage protein (NRAMP) proteins^74^; As moves in rice via silicon channels and NIP proteins. Pb has weak mobility in plants, often fixed in root cell walls and vacuoles^75^; essential elements like Zn, Cu, Ni enter phloem toward grains via YSL, ZIP proteins. Vacuolar sequestration and chelate formation are key detoxification strategies. Glutathione, phytochelatins (PCs), and metallothioneins (MTs) can complex metals, reducing their toxicity.

### Molecular mechanisms of heavy metal transport in plants

After entering root cells, heavy metals are transported via xylem and phloem, with their distribution governed by specialized transmembrane transporters (Cao et al., 2020).^73^ Root uptake of divalent cations (e.g., Zn^2+^, Fe^2+^, Cd^2+^) is primarily mediated by ZIP/IRT1 and NRAMP families. IRT1, responsible for iron acquisition, also facilitates Cd^2+^ entry due to broad substrate specificity; its upregulation under iron deficiency inadvertently increases Cd uptake. OsNRAMP5 in rice contributes >80% of root Cd influx, making it a key target for breeding low-Cd varieties.^74^ Following uptake, HMA proteins direct metal translocation: HMA2/HMA4 load Zn and Cd into the xylem for shoot transport, while HMA3 sequesters metals into root vacuoles, preventing upward movement. Allelic variation in OsHMA3 is linked to differential Cd accumulation among rice cultivars.

For As, the Lsi (low silicon) channels (Lsi1 and Lsi2) constitute the primary entry pathway; arsenite (As (III)) is transported inadvertently due to structural similarity with silicic acid, explaining rice’s high susceptibility to As contamination.^41^ YSL (yellow stripe-like) transporters facilitate phloem-mediated redistribution of metal-phytosiderophore complexes (e.g., Fe, Ni, etc.) to grains, while ABC (ATP-binding cassette) transporters sequester metal-chelate complexes into vacuoles. PCs and MTs bind metals such as Cd, As, and Hg, with the resulting complexes transported into vacuoles by ABC transporters to reduce free metal ion toxicity.^76^ The coordinated action of these families determines net metal accumulation; for Cd, the pathway involves root uptake (IRT1/NRAMP5), vacuolar sequestration (HMA3), xylem loading (HMA2/HMA4), and phloem redistribution to grains (YSL). This molecular framework provides targets for marker-assisted breeding and gene editing (e.g., CRISPR-Cas9 targeting OsNRAMP5) to develop low-accumulation crop varieties. Table S8 summarizes the major transporter families and their functional roles.

Different crops exhibit significant variations in heavy metal accumulation capacity and distribution characteristics. Rice has a strong capacity to accumulate As and Cd^77^; leafy vegetables are sensitive to Pb and Zn; root vegetables can accumulate more metals in their cortex. Crop organ structure, metabolic demands, and cultivation conditions influence accumulation sites. Flooded paddy conditions promote As and Cd migration, posing significant grain risks^78^; leafy vegetables, with large surface areas and strong transpiration, accumulate Pb, Zn, and Cd notably in leaves; peeling root vegetables (carrot, potato, radish, etc.) effectively reduces intake risk as metals deposit mainly in the cortex. Cd and Zn easily migrate to grains of cereals like corn and wheat; long-term consumption may affect health. Heavy metal content in fruits of solanaceous crops (tomato, eggplant, etc.) is relatively low, but leaves and stems contain higher levels.^79^

Recent functional genomics studies have identified key molecular targets for breeding low-Cd crops. Hassan et al. employed CRISPR-Cas9 knockout approaches to validate the role of *SlNRAMP3* in Cd uptake and translocation in tomato. Their findings demonstrate that loss-of-function mutations in this transporter reduce Cd accumulation in fruits by approximately 60% without compromising yield or essential nutrient content. This study provides a proof-of-concept for targeted gene editing as a viable strategy for reducing dietary Cd exposure, with implications for staple crops such as rice and wheat.^80^

## Hazards of heavy metals to crops and molecular stress mechanisms

### Macroscopic hazards to crops

Heavy metal stress significantly impacts crop growth and development, manifesting as reduced seed germination rate, inhibited root growth, leaf chlorosis, plant stunting, and yield reduction.^81^ Some metals (e.g., Cd, Pb, Hg, Cr, etc.) severely damage plant physiological metabolism by disrupting cell membranes and photosynthetic systems. High concentrations can interfere with various metalloenzyme functions by competing with essential metals (e.g., Zn, Fe, Mg, etc.) for binding sites.^82^ For instance, Cd displaces Zn in enzyme proteins, causing inactivation; Cr(VI)’s strong oxidizing property can destroy membrane lipids, generating a large amount of reactive oxygen species (ROS).^28^ Studies show that at Cd concentrations of 5–10 mg/kg, wheat root length and plant height significantly decrease, chlorophyll content drops over 40%; Pb and Hg stress can inhibit photosystem II (PSII) electron transport, reducing photosynthetic rate by over 30%.^83^ Accumulation in cell walls, vacuoles, and root epidermis leads to abnormal root morphology, affecting nutrient uptake and water transport, key reasons for reduced crop yield and quality. The impact of heavy metal stress on growth and yield of major crops is shown in Table S9.

#### Growth and development inhibition

Heavy metals can inhibit seed germination, root elongation, and plant height increase in crops, leading to reduced biomass.^81^ For example, Cd can damage the cellular structure of rice roots, reducing the root absorption area^84^; Pb can inhibit photosynthesis in wheat, resulting in leaf chlorosis and decreased tillering. Studies have shown that when soil Cd concentration reaches a certain level, the germination rate of rice seeds is significantly reduced, root growth is inhibited, and root length is shortened by 30%–50% compared to normal conditions. In lead-contaminated soil, chlorophyll content in wheat leaves decreases significantly, photosynthetic rate declines, consequently leading to stunted plants, reduced tiller numbers, and severely impacting both yield and quality.

#### Yield and quality decline

Heavy metal pollution leads to reduced seed setting rate (e.g., increased barren stalks in corn), unfilled grains, while decreasing protein, vitamin content, and increasing toxic substance accumulation (e.g., “cadmium rice” has reduced starch content). Corn grown in Cd-polluted fields has a barren stalk rate 20%–30% higher than unpolluted fields, with poor grain plumpness and lower thousand-kernel weight. Furthermore, nutrient composition changes in heavy metal-polluted crops; for example, “cadmium rice” has reduced starch content, and protein structure may alter, lowering nutritional value,^85^ while toxic substance (e.g., heavy metals themselves) accumulation increases food safety risks.

### Molecular stress mechanisms

Heavy metals can induce excessive production of ROS, leading to oxidative stress in plants.^86^ Plants regulate redox homeostasis through antioxidant enzyme systems such as superoxide dismutase (SOD), catalase (CAT), and peroxidase (POD), as well as non-enzymatic antioxidants like glutathione (GSH). By inhibiting the mitochondrial electron transport chain and the chloroplast photosynthetic chain, heavy metals cause the accumulation of superoxide anion (O_2_^−^·), hydrogen peroxide (H_2_O_2_), and hydroxyl radical (·OH), thereby inducing membrane lipid peroxidation and cellular damage. Research indicates that under Cd stress, the malondialdehyde (MDA) content in wheat leaves increases by 2–3 times, reflecting aggravated membrane damage^87^; the activities of SOD and CAT are upregulated in the short term but decline under prolonged high-concentration exposure, indicating gradual depletion of the plant’s antioxidant defense capacity.^88^ The ascorbate-GSH (ASA-GSH) cycle, composed of GSH and ASA, serves as the central pathway for ROS scavenging. PCs, synthesized using GSH as a substrate, form complexes with metals such as Cd, As, and Hg and transport them to vacuoles for sequestration, thereby reducing the toxicity of free metal ions while maintaining cellular homeostasis.

Recent transcriptomic and proteomic studies have significantly advanced the understanding of plant molecular responses to heavy metal stress. In *Arabidopsis thaliana*, integrated multi-omics analyses revealed that Cd, Cu, and Zn stress induce distinct alterations in fatty acid and sugar metabolism, with parallel proteomics identifying over 1,200 differentially expressed proteins under Cd stress alone, predominantly linked to photosynthesis, oxidative stress responses, and redox-related enzymatic functions; notably, while SOD activity displayed metal-specific responses, CAT and POD activities were broadly suppressed across multiple metal treatments.^89^ In rapeseed, transcriptome profiling under Cd, Pb, and As stress highlighted the central roles of POD-encoding genes, ABC transporters, and abscisic acid signaling pathways in coordinating stress adaptation. Furthermore, cell wall remodeling has emerged as a critical defense layer, with the xyloglucan endotransglucosylase/hydrolase gene *RsXTH25* in radish being strongly induced by Pb stress; overexpression of this gene enhances Pb tolerance by reducing oxidative damage and maintaining photosynthetic performance.^90^

Beyond transcriptional regulation, epigenetic and post-transcriptional mechanisms play pivotal roles in heavy metal adaptation. Cadmium stress in rice downregulates miR166 expression, which in turn targets *OsHB4* to modulate Cd accumulation and translocation; overexpression of miR166 enhances Cd tolerance while reducing grain Cd content. MAPK signaling cascades and phytohormonal crosstalk, particularly involving abscisic acid, integrate stress signals to activate downstream transcription factors such as WRKY, MYB, and NAC families, which orchestrate the expression of antioxidant enzymes and metal chelators.^91^ Recent evidence also indicates that heavy metal exposure induces dynamic changes in DNA methylation patterns, with hypomethylation at promoter regions of metal resistance genes correlating with their elevated expression, suggesting an epigenetic basis for transgenerational stress memory. These molecular insights collectively provide a framework for understanding how plants sense, respond to, and adapt to heavy metal toxicity at multiple regulatory levels. Table S10 summarizes the characteristics of plant antioxidant system responses under different heavy metal stresses.

#### Oxidative stress damage

Heavy metals (e.g., Cd, Cu, etc.) can induce the excessive generation of ROS (e.g., superoxide anion and hydrogen peroxide) in crop cells, disrupting the intracellular oxidant-antioxidant balance and resulting in membrane lipid peroxidation, protein denaturation, and DNA damage. When crops are subjected to Cd stress, the activities of intracellular antioxidant enzymes (e.g., SOD, CAT, etc.) undergo dynamic changes.^92^ Initially, SOD activity increases to scavenge excess superoxide anions. However, as Cd exposure prolongs and intensifies, the antioxidant enzyme system becomes progressively imbalanced, leading to substantial accumulation of ROS such as superoxide anions and hydrogen peroxide.^93^ These ROS attack unsaturated fatty acids in the cell membrane, triggering lipid peroxidation, which compromises membrane structure and function, induces leakage of intracellular contents, and disrupts normal cellular metabolism.

#### Enzyme activity interference

Heavy metals can bind to enzyme active sites (e.g., thiol, carboxyl groups, etc.), inhibiting key enzyme activity. For example, Cd can inhibit Rubisco enzyme in photosynthesis, reducing carbon assimilation efficiency^94^; Pb can inhibit succinate dehydrogenase in respiration, affecting energy metabolism. Rubisco is the key enzyme for CO_2_ fixation in photosynthesis. Cadmium binding to its active site alters enzyme conformation, reducing affinity for CO_2_,^95^ thereby decreasing carbon assimilation efficiency and photosynthetic product synthesis. Succinate dehydrogenase plays a vital role in the TCA cycle of respiration. Lead inhibition of this enzyme obstructs respiratory chain electron transport, reducing cellular energy production, affecting crop growth and development.^96^

#### Genetic material damage

Heavy metals (e.g., As, Cr, etc.) can cause DNA strand breaks, base mutations, or chromosome aberrations in crops, interfering with gene replication and expression, thereby affecting cell division and organ development. Studies find that As-treated *Arabidopsis* plants show a large amount of DNA strand breaks in root tip cells,^97^ alongside significant changes in gene expression profiles, with many genes related to cell division and growth/development being suppressed. Chromium pollution also causes chromosome aberrations in crops, like breaks, deletions, translocations.^98^ Such genetic damage leads to abnormal growth, deformed seedlings, and stunted plants.

#### Signal transduction and hormonal disruption

Heavy metals can interfere with the synthesis and transport of hormones (e.g., auxin, cytokinin, etc.) in crops, disrupting signal transduction pathways, leading to imbalanced growth regulation. For example, Cd can inhibit polar auxin transport, causing the obstruction of root growth.^99^ Auxin plays a crucial regulatory role in plant growth and development; its polar transport is essential for maintaining normal organ morphology and function.^100^ Under Cd stress, the activity of auxin transport carrier proteins is inhibited, causing abnormal auxin distribution in areas like root tips, subsequently affecting root tip cell elongation and division, ultimately manifesting as inhibited root growth, reduced lateral roots, and altered root morphology.^101^

### Signal pathways and metabolic interference

**Figure 2:**
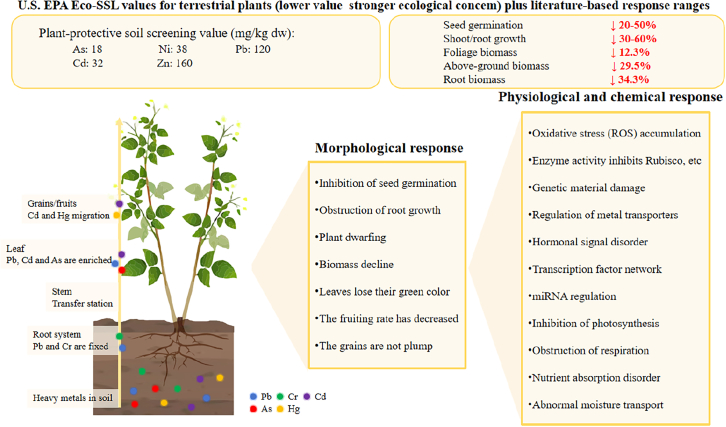
Effects of heavy metals on plants and plant response mechanisms (Image source, drawn by the author).

As shown in Figure 2, plants implement defense responses under heavy metal stress through various signaling pathways and transcriptional regulation, including metal transporter regulation, chelator synthesis, and ROS signaling networks.^102^ Heavy metal stress often causes nutrient element imbalance in plants. For instance, Cd can compete with Zn and Fe for binding sites, inhibiting chlorophyll synthesis; Pb can inhibit calcium ion signaling, affecting cell wall structure; Hg competes with selenium for sulfur groups, disrupting selenoenzyme function.^103^ Research shows that at Cd concentration of 5 mg/kg, Fe and Mn content in wheat decreases over 30%; Cr stress reduces Mg content, affecting photosynthetic efficiency. Nutrient imbalance exacerbates plant sensitivity to environmental stress.

Metal Transporter Regulation: The HMA family regulates Cd and Zn transport to shoots; NRAMP proteins participate in Mn, Cd, and Fe transmembrane transport; Lsi channels determine As uptake in rice; ABC transporters function in metal compartmentalization and vacuolar storage.^104^

Hormonal signals, hormones like ABA, JA, and SA enhance plant tolerance by regulating stomatal closure and antioxidant enzyme expression under metal stress; ethylene can regulate root morphology in response to Cd stress.

Transcription factor networks, transcription factors like WRKY, bZIP, MYB, and NAC can activate metal chelator synthesis genes (e.g., PCS1), enhancing detoxification capacity.^105^

miRNA Regulation, miR398, miR395, etc., participate in regulating metal-induced oxidative stress and sulfur metabolism gene expression, helping plants rebuild metabolic networks.

Heavy metals affect crop growth through ion competition, oxidative stress, signal regulation, and gene expression at multiple levels,^106^ leading to the obstruction of growth, yield reduction, and quality decline. Plants alleviate metal toxicity through strategies like cell wall fixation, chelation, vacuolar sequestration, antioxidant systems, and transcriptional regulation.^76^ However, under high concentration exposure, these mechanisms often fail, causing severe dietary risks. In-depth analysis of these response mechanisms not only provides a theoretical basis for agronomic control and soil remediation but also offers gene targets for breeding low-accumulation, heavy metal-tolerant crop varieties.

An emerging concern in environmental health is the co-selection of antimicrobial resistance (AMR) by heavy metal pollution. Sánchez-Corona et al. provide a comprehensive analysis of how heavy metals such as Cu, Zn, and Cd exert selective pressure on microbial communities, promoting the co-transfer of metal resistance genes and antibiotic resistance genes via mobile genetic elements.^107^ This co-selection mechanism is particularly relevant in agricultural soils receiving livestock manure, where high Cu and Zn concentrations create hotspots for AMR dissemination. The study highlights the need for integrated management strategies that address both heavy metal pollution and the emerging threat of AMR in agroecosystems.

**Figure 3:**
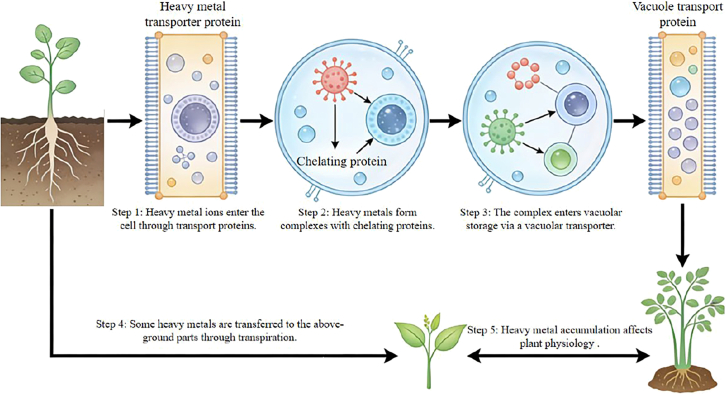
Schematic diagram of heavy metal absorption, transport, and toxic effects in plant cells (Image source, drawn by the author).

As shown in Figure 3, for combined pollution, the joint toxicity mechanism extends beyond simple additive effects, requiring integration of toxicology and plant physiology. Co-occurring metals can exhibit antagonistic interactions—for instance, Cd and Zn compete for shared transporters (IRT1 and ZIP), reducing Cd accumulation when Zn is abundant—or synergistic effects—As and Cd in flooded paddies show contrasting mobilization patterns, with As (III) increasing while Cd decreases, complicating mitigation. At the physiological level, multiple metals can trigger oxidative stress through distinct but overlapping pathways: Cd disrupts mitochondrial electron transport, Cr (VI) directly generates ROS via reduction, and Pb inhibits antioxidant enzymes. This complexity necessitates a shift from single-metal risk assessment to multi-metal interactive frameworks. Emerging approaches, such as integrated biomarker responses and mixture toxicity models (e.g., concentration addition, independent action, etc.), offer quantitative tools to evaluate combined risks, while omics technologies (transcriptomics, metabolomics, etc.) are beginning to unravel the molecular networks underlying metal-metal interactions. Signaling molecules and regulatory factors associated with heavy metal stress are listed in Table S11.

## Pathways and hazards of heavy metals entering humans via crops

### Pathways of heavy metals entering humans via crops

Heavy metals in soil enter the human body primarily through the food chain after being absorbed and accumulated by crops, constituting a significant environmental health risk.^108^ Crop absorption capacity for heavy metals is influenced by soil physicochemical properties (e.g., pH, organic matter content, CEC, etc.), heavy metal type, and chemical speciation.^109^ For instance, Cd solubility is higher in acidic soil, enhancing rice Cd uptake; whereas Pb mostly exists in insoluble forms in soil but can still be partially absorbed under rhizosphere acidification.

#### Grain crop pathway

Grain crops are the main energy source for humans. Rice, wheat, corn, etc., have a certain capacity to absorb and accumulate heavy metals from soil. Studies show that rice grains in high-Cd soil can accumulate Cd up to 0.1–1.0 mg/kg (dry weight), far exceeding some food safety limits (e.g., Chinese standard GB2762-2022 sets rice Cd limit at 0.2 mg/kg). When people consume contaminated rice and wheat products, heavy metals enter the body with food, accumulating in vital organs like the liver and kidneys, impacting health long-term.^108^

#### Vegetable and fruit pathway

Vegetables and fruits are high-risk sources of heavy metal exposure due to direct consumption. Leafy vegetables (e.g., spinach, rapeseed, etc.) and root vegetables (e.g., carrot, potato, etc.) show strong accumulation capacity for Cd, Pb, Cu, etc.^79^ Research indicates Cd in spinach leaves can reach 0.5–1.5 mg/kg, Pb in radish roots can accumulate to 0.2–0.8 mg/kg. Daily intake of these vegetables allows heavy metals to enter the bloodstream via digestive tract absorption, potentially causing chronic poisoning, renal damage, or bone metabolism abnormalities.^110^ Although heavy metal content in fruits is relatively low, long-term consumption of contaminated fruits (e.g., apples, bananas, etc.) still increases cumulative risks of Cd, Pb, Hg, etc.

#### Processed food pathway

Processed crop-based foods are also important pathways for heavy metals entering humans.^111^ Flour, pastries from contaminated wheat, or jam, juice, dried fruits from contaminated fruits may retain original heavy metals. Rice noodles processed from Cd-contaminated rice can have Cd content 70%–90% of the original grain; long-term consumption significantly increases human Cd intake.

#### Composite foods and hidden exposure

In modern diets, people often consume composite foods made from various crops, like instant noodles, breakfast cereals, vegetable salads, which can be “hidden” sources of heavy metal exposure. Research finds that long-term consumption of contaminated produce from multiple sources, even if individual item levels are not exceeding the standard, may lead to cumulative exposure exceeding safety thresholds.

#### Risks for children and sensitive populations

Children are more sensitive to heavy metals due to rapid growth, development, and high intestinal absorption rates.^112^ Pb, Cd, As, etc., accumulated in soil and crops easily enter children’s bodies via consumed rice, vegetables, juice, adversely affecting the nervous system, renal function, and bone development.^113^ Heavy metal intake via food by pregnant women can also affect the fetus through the placental barrier, increasing risks of low birth weight and premature birth.

In summary, the main pathways for heavy metals entering humans via crops include direct consumption of contaminated grains, vegetables, fruits, as well as cumulative and composite exposure in processed foods. Different crop types, soil properties, processing methods, and consumption habits influence heavy metal absorption and accumulation levels in humans. Therefore, scientifically assessing the soil-crop-human heavy metal transfer pathway is crucial for establishing food safety standards and conducting risk management.

#### Quantitative risk assessment approaches

To translate heavy metal concentrations in crops into actionable health risk estimates, quantitative indices are widely employed. The hazard quotient (HQ) is calculated as the ratio of estimated daily intake to the reference dose (RfD); an HQ > 1 indicates potential non-carcinogenic risk (non-CR). The hazard index (HI) sums HQs for multiple metals to assess cumulative risk from co-exposure. For CRs, the CR is estimated by multiplying lifetime average daily intake by the cancer slope factor, with a CR > 1.0 × 10^−4^ typically considered unacceptable. Using the Cd levels in rice reported in Section 2.1 (0.1–1.0 mg/kg), the HQ for a typical adult consuming 300 g rice daily would range from 0.5 to 5.0, indicating potential health concerns in high-exposure scenarios.^114^ These quantitative frameworks enable consistent comparison across studies and support evidence-based food safety standards and risk communication. Table 4 provides an overview of heavy metal accumulation in crops and associated human health risks.

**Table 4:** Accumulation of heavy metals in crops and associated human health risks

Heavy metal	Main accumulating crops	Typical accumulation content (mg/kg, dry weight)	Main human health effects	Remarks
**Cd**	Rice, spinach, corn	0.1–1.5	Renal damage, bone metabolism abnormalities, chronic poisoning	Rice grains particularly prone to Cd accumulation
**Pb**	Radish, rapeseed, tomato	0.05–0.8	Nervous system damage, child developmental disorders, elevated blood pressure	Leafy and root vegetables accumulate more
**As**	Rice, corn	0.05–0.5	Skin lesions, cancer, fetal developmental abnormalities	As mainly exists as inorganic arsenic in rice grains
**Hg**	Rice, Vegetables, Fruit trees	0.01–0.2	Neurotoxicity, renal damage, immune suppression	Significantly affected by industrial pollution areas
**Cu**	Vegetables (spinach, leek), fruits	1–15	Liver damage, gastrointestinal discomfort	Crops have a relatively strong ability to absorb Cu, but they generally do not exceed the standard
**Zn**	Corn, wheat, vegetables	10–50	GI discomfort, long-term excess affects Cu absorption	Essential trace element, but excess also risky

Note, the table and all related contents were compiled by the author.

### Target organs and hazards of heavy metal toxicity

#### Cadmium

**Figure 4:**
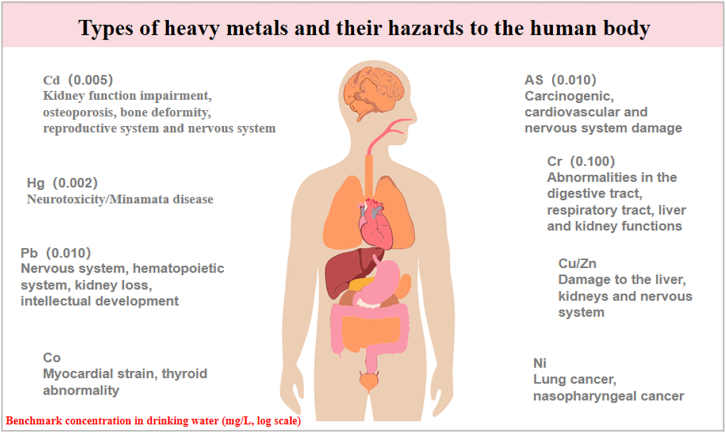
Main hazards of different heavy metals to the human body (Image source, drawn by the author).

As shown in Figure 4, Cadmium primarily accumulates in the kidneys and liver, with a biological half-life of 10–30 years, resulting in strong chronic toxicity.^115^ Chronic exposure typically causes renal tubular dysfunction, characterized by proteinuria, as well as osteoporosis and osteomalacia due to Cd-induced disruption of calcium metabolism.^116^ The classic “Itai-itai disease” in Japan resulted from long-term consumption of Cd-contaminated rice, with affected patients presenting severe bone pain and pathological fractures; epidemiological studies reported fracture rates exceeding 65% among female patients in the Jinzu river basin. Mechanistically, Cd induces renal damage by triggering oxidative stress and apoptosis in proximal tubular cells, while interfering with Ca, Fe, and Zn metabolism, leading to secondary anemia and immune suppression.^117^ Cd is classified as a group 1 carcinogen by the IARC, with evidence linking chronic exposure to lung, prostate, and kidney cancers.

#### Lead

Lead is particularly harmful to children. Its main target organs are the nervous system, hematopoietic system, and kidneys.^118^ For every 10 μg/dL increase in child blood Pb level, IQ decreases by 1–2 points on average; WHO reports approximately 800 million children globally have blood Pb levels ≥50 μg/L. Adult Pb exposure manifests as anemia, hypertension, neurasthenia, and glomerulosclerosis.^119^ Occupational exposure (battery manufacturing, smelting workers, etc.) often shows symptoms like “lead lines” on gums.

#### Mercury

Mercury toxicity varies by species. Methylmercury is most toxic, easily crossing the blood-brain and placental barriers, severely harming the nervous system and fetal development.^120^ Typical Minamata disease patients exhibit sensory disturbances, ataxia, speech impairment,^121^ hearing loss, even congenital malformations in infants. WHO estimates that over 15 million people are threatened by Hg pollution annually, most prominently fish consumers. Inorganic Hg mainly damages kidneys, causing proteinuria and renal tubular necrosis.^122^

#### Arsenic

**Figure 5:**
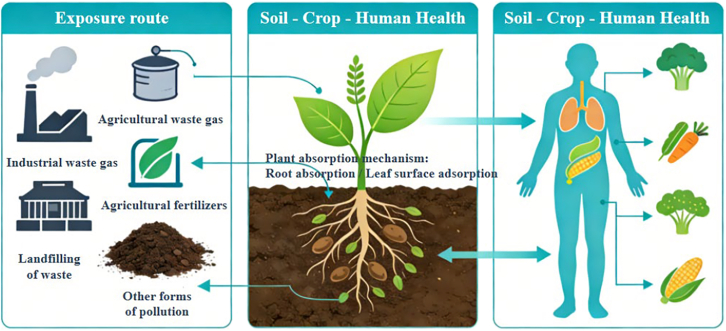
“Soil-crop-human health” exposure pathway (Image source, drawn by the author).

As shown in Figure 5, Arsenic exposure is most severe via drinking water. Long-term inorganic As intake leads to skin lesions (keratosis, pigmentation), cardiovascular diseases, and various cancers.^123^ In Bangladesh and West Bengal, India, over 40 million people drink high-As water, with skin lesion prevalence reaching 15%–20%. Chronic arsenicism is closely related to skin, bladder, and lung cancer. Mechanistically, As promotes tumorigenesis through oxidative stress and DNA methylation abnormalities.^124^

#### Chromium

Hexavalent Cr (Cr (VI)) is a strong oxidant, far more toxic than trivalent Cr (Cr (III)).^125^ Its target organs are the respiratory system, liver, and skin. Long-term occupational exposure can cause nasal septum perforation, chronic bronchitis, lung cancer. Studies show standardized lung cancer incidence rates among electroplating workers increase 3–5 times. Cr (VI) exerts carcinogenic effects by generating ROS and causing DNA damage.^126^

#### Copper

Copper is an essential element, but excess causes liver and nervous system damage.^127^ The typical disease is Wilson’s disease (hereditary Cu metabolism disorder), with patient cirrhosis rate over 50%, accompanied by neurological and psychiatric abnormalities. Environmental Cu exposure (e.g., exceeding the standard drinking water Cu) can cause gastroenteritis, liver dysfunction. Animal experiments show long-term high-Cu diet increases rat liver lipid peroxidation by over 60%.

#### Zinc

Zinc is an essential element, but excessive intake is also toxic. Acute Zn poisoning causes nausea, vomiting, abdominal pain^128^; chronic high-dose exposure inhibits Cu and Fe absorption, leading to anemia,^129^ immune dysfunction, and neurological damage. Studies show anemia incidence increases over 20% among residents drinking high-Zn water (>10 mg/L) for a long time. Table S12 summarizes the target organs and health hazards of major heavy metals.

## Early warning measures for heavy metal pollution

### Source monitoring

Source monitoring of soil heavy metal pollution is the primary step to block exposure, requiring focus on the sources and occurrence states of heavy metals in soil to identify high-risk polluted areas.^130^ For industrial pollution sources, a soil monitoring network around industrial parks should be established, using systematic layout and high-frequency sampling strategies, combined with ICP-MS technology for regular determination of total and bioavailable content of Cd, Pb, Hg, etc., focusing on farmland soil within 5 km of enterprises like electroplating and smelting, assessing pollutant dispersion range and intensity. Agricultural source monitoring needs to focus on fertilizer, pesticide application, and sewage irrigation areas, judging heavy metal bioavailability by analyzing soil pH, organic matter content, and heavy metal speciation—for example, higher Cd activity in acidic soil requires marking as high environmental health risk areas. Differentiating between natural and anthropogenic sources is also crucial. Techniques like Pb isotope ratio analysis can trace whether soil Pb originates from industrial emissions or natural weathering, providing evidence for precise control.^131^ For soil with monitoring data exceeding standards, environmental health risk control areas should be delineated, prohibiting edible crop planting, recommending switching to energy crops or flowers (non-food chain crops), cutting off exposure pathways at the source.

### Process monitoring

Tracking the dynamic risk of crop uptake and accumulation is the core link connecting soil pollution and population exposure, requiring focus on heavy metal accumulation patterns in different crop organs and key growth stages. For grain crops like rice and wheat, samples of roots, leaves, and grains should be collected at key stages like seedling and grain filling, detecting changes in heavy metal content, clarifying transfer efficiency of Cd, As, etc., to edible parts. Studies show over 60% of Cd transfer to rice grains occurs during the grain filling stage,^132^ necessitating intensified monitoring. Leafy vegetables (e.g., spinach, lettuce, etc.) have strong Pb, Cd accumulation capacity. *In situ* monitoring techniques like portable X-ray fluorescence (XRF) analyzer should be used for real-time leaf heavy metal monitoring, combined with soil pollution data to establish “soil-crop” transfer models predicting edible part超标 risk. For root crops (e.g., potato, carrot, etc.), focus on the accumulation factor of soil heavy metals in tubers; when the Cd accumulation factor exceeds 0.5, it indicates high environmental health risk. Additionally, key environmental factors affecting heavy metal uptake need monitoring, e.g., soil organic matter can reduce heavy metal bioavailability, while low pH promotes Cd, Zn absorption.^109^ Dynamically recording these parameters optimizes risk assessment accuracy.

### Population health monitoring

Assessing the terminal health effects of heavy metal exposure is the final link in environmental health risk assessment, requiring quantification of potential harm through biomarker detection and epidemiological investigation. For residents in high-risk areas, blood and urine samples should be collected regularly to detect biomarkers like blood Pb and urinary Cd^133^—when child blood Pb exceeds 50 μg/L, intervention measures should be initiated; adult urinary Cd over 5 μg/g creatinine indicates increased renal damage risk. Epidemiological investigations should focus on assessing the health impacts on exposed populations, such as whether the incidence of hypertension and chronic kidney disease among people who have long consumed crops with excessive heavy metals is higher than that in the control population. Through logistic regression analysis, the dose-response relationship between heavy metal exposure and health effects should be clarified. Simultaneously, special monitoring for sensitive populations (children, pregnant women, and elderly), analyzing the correlation between child neurodevelopmental indicators (e.g., IQ score, attention tests) and blood Pb levels can directly reflect the severity of environmental health risks.^134^

### Full-chain risk assessment

Based on monitoring data from “source-process-population,” a tiered risk assessment model should be used: Step 1 calculates pollution index based on soil heavy metal content, classifying pollution levels; Step 2 combines crop accumulation factors to calculate dietary exposure, e.g., if adult daily Cd intake via rice exceeds 0.8 μg/kg body weight, health risk exists; Step 3 assesses actual health damage risk through biomarker exceeding the standard rates and health effect incidence. Risk assessment results should translate into specific control measures: implement soil remediation (e.g., applying lime to reduce Cd activity) in very high-risk areas; promote low-accumulation crop varieties (e.g., “Xiangzaoxian 45” rice) in medium-risk areas; strengthen agricultural product sampling in low-risk areas. Simultaneously, establish a risk early warning system; when monitoring data triggers thresholds (e.g., vegetable Pb content >0.1 mg/kg), promptly issue consumption alerts to block exposure pathways.

Build a national soil heavy metal pollution monitoring network, regularly assessing soil quality and crop heavy metal content; conduct health risk assessments in polluted areas, delineate risk control areas, restrict circulation of contaminated agricultural products. Utilize modern IT (e.g., internet of things [IoT], remote sensing) to construct a soil heavy metal pollution monitoring network for real-time monitoring and dynamic early warning of soil heavy metal content. Regularly collect and analyze soil and agricultural product samples, clarifying pollution degree and safety risks, enforce mandatory testing of products from risk areas, ban exceed the standard products from the market, conduct health education and population monitoring, enhance public protection awareness.

The threat of metal-polluted soil to environmental health is concealed and persistent. Integrating resident health detection into environmental health management practices, from early risk warning to optimized control measures and social participation, builds an “environment-crop-population” health protection closed loop by converting soil pollution environmental risk into quantifiable health data. This process embodies the essence of environmental health centered on population health and promotes the transition of heavy metal pollution control from simple soil remediation to synergistic “environmental restoration-health protection.” Continuously strengthening the core role of resident health detection in environmental health management, deeply integrating it with soil remediation, agricultural product regulation, and health interventions, can systematically block the heavy metal health hazard chain, providing solid guarantee for achieving the environmental health goals of “soil safety, food safety, population health,” ultimately promoting sustainable coexistence of humans and the environment.

In recent years, the integration of artificial intelligence and advanced sensing technologies has significantly enhanced the forward-looking capabilities of heavy metal pollution monitoring and risk warning. In terms of source analysis and risk prediction, machine learning models (such as random forests, gradient boosting) have been successfully applied to distinguish between natural background and anthropogenic pollution sources, while deep learning algorithms combined with satellite remote sensing and soil attribute data can achieve high-precision mapping of pollution hotspots^135^^,^^136^; in the prediction of biological effectiveness, support vector machines and other models, through training parameters such as soil pH and organic matter, can quantitatively predict the accumulation of heavy metals in crops (such as rice), with a prediction accuracy (R^2^) exceeding 0.85, providing scientific basis for field management.^137^ In the field of real-time monitoring, portable XRF analyzers have enabled on-site rapid screening of soil and crops, while unmanned aerial vehicles equipped with hyperspectral sensors can non-destructively infer the content of heavy metals in crops through leaf reflectance spectra, with a prediction accuracy of over 80%.^138^^,^^139^ The collaborative application of these technologies marks a paradigm shift in heavy metal risk management from passive response to active warning, and from single-point monitoring to real-time intelligent monitoring. Full-chain monitoring indicators and risk thresholds are detailed in Table S13.

### Limitations of monitoring technology

Despite the promise of these advanced monitoring technologies, several limitations currently constrain their widespread field application. First, high equipment and deployment costs remain prohibitive: hyperspectral sensors, IoT networks, and portable XRF analyzers require substantial capital investment, limiting their accessibility to well-funded research institutions rather than routine agricultural monitoring. Second, data standardization and interoperability challenges persist; monitoring data generated by different sensor platforms often lack unified formats and quality control protocols, hindering integration into centralized early warning systems. Third, the interpretability and transferability of AI models pose scientific challenges: machine learning predictions trained on site-specific datasets frequently fail to generalize across regions with different soil types, climate regimes, and agricultural practices. Furthermore, the “black box” nature of deep learning models limits mechanistic understanding of underlying environmental processes, which is essential for building trust among regulators and land managers. And fourth, the long-term stability and field validation of sensor-based technologies remain understudied; most validation studies are conducted under controlled conditions rather than realistic field scenarios with variable weather, crop rotations, and management practices. Addressing these limitations requires interdisciplinary collaboration among engineers, data scientists, agronomists, and environmental regulators to develop cost-effective, interoperable, and interpretable monitoring systems that can transition from research prototypes to operational tools.

## Prevention and control measures for heavy metal pollution

### Source control

#### Strict control of pollution sources

Heavy metal pollution prevention should adhere to the principle of “prevention first, source control.” Formulate and strictly enforce industrial wastewater and air emission standards,^140^ promote clean production technologies, completely eliminate the extensive “pollute first, control later” model. For example, China implemented the “Electroplating Pollutant Discharge Standard” for industries like electroplating and smelting, clearly stipulated the maximum allowable discharge concentrations of Cd, Cr, Pb, As, etc., in wastewater. Electroplating enterprises should undergo mandatory clean production audits, promote advanced wastewater treatment technologies like efficient membrane separation and electrolytic recovery, ensuring meet the standards discharge. In agriculture, standardize fertilizer and pesticide use, and completely ban As-containing agents (e.g., glyphosate-arsenate herbicides) and highly toxic pesticides containing Pb and Hg.^141^ Establish fertilizer and pesticide use registration and traceability systems, promote formulated cc, minimizing agricultural heavy metal input.

#### Optimize industrial and agricultural structure

For areas with severe soil pollution, fundamentally reduce heavy metal input pressure through industrial transformation, upgrading, and layout adjustment.^49^ On one hand, relocate or technically renovate heavily polluting enterprises like smelting, chemicals, leather, especially those near cities or densely populated areas, implementing relocation to avoid continued pollutant accumulation in surrounding soil. On the other hand, reasonably adjust planting structure in polluted areas, avoiding food crop contamination. For lightly polluted areas, develop non-edible economic crops or energy crops (e.g., *Jatropha*, rapeseed, etc.), which can adapt to heavy metal stress and achieve bioenergy production through oil extraction, attaining “ecological-economic” dual benefits. Medium and severe polluted areas should withdraw from food production and turn to the development of non-food chain industries such as ornamental flowers and seedlings, completely block the way of heavy metals entering the food chain through crops, and realize the safe and sustainable use of polluted farmland.^142^

### Contaminated soil remediation technologies

Remediation of heavy metal-contaminated agricultural soils encompasses a range of approaches, each with distinct advantages, limitations, and applicability to field conditions. Phytoremediation, which utilizes hyperaccumulators such as *Pteris vittata* (As) and *Sedum alfredii* (Cd), offers low cost and ecological compatibility, with As accumulation factors reaching 10–20 in ferns. However, its primary limitations include long remediation timelines (often requiring 5–15 years), shallow treatment depth (typically confined to topsoil), and climate-dependent performance, making it more suitable for light to moderate contamination in rural settings where time is not the primary constraint. Chemical immobilization—through amendments like lime, phosphate, biochar, or Fe/Mn oxides—reduces metal bioavailability rapidly (within a single growing season) and is cost-effective for large areas. Yet, it does not remove metals from soil but rather converts them to less soluble forms; its long-term stability under changing environmental conditions (e.g., soil acidification, flooding, etc.) remains uncertain, requiring careful monitoring. Nanomaterial-based remediation (e.g., nano-zero-valent iron, nano-hydroxyapatite, etc.) exhibits high efficiency, with studies showing >70% reduction in available Cd or Cr(VI) within days, but faces challenges including high cost, potential ecotoxicity, and risk of aggregation, limiting its current application to small-scale hot spots rather than widespread field use. Soil washing and electrokinetic remediation achieve rapid removal of total metals (50%–90% efficiency) but are costly, disruptive to soil structure, and generate secondary waste, making them suitable primarily for industrial legacy sites rather than active agricultural land. Integrated approaches combining chemical immobilization with agronomic measures (e.g., water management and low-accumulation cultivars) are increasingly adopted as a balanced strategy, offering both short-term risk reduction and long-term sustainability. For instance, liming coupled with intermittent flooding has been shown to reduce grain Cd by 40%–60% while maintaining yield, representing a practical trade-off between efficacy, cost, and ecological safety. Table 5 provides a comparative summary of these technologies.

**Table 5:** Comparison of different remediation technologies for metal contaminated soil

Remediation category	Representative method	Basic principle	Advantages	Disadvantages	Applicability	Remediation Efficiency
**Physical**	Soil replacement	Replace polluted soil with clean soil	Fast results, intuitive effects	High cost, needs a large amount of imported soil	Severe and localized pollution	Pollutant concentration reduction >80%
–	Electrokinetic remediation	Electric field drives metal migration	Suitable low-permeability soil, selective	High energy, long cycle	Clay soil, Cr, Cd pollution	Cd removal 60%–70%
**Chemical**	Soil washing	Chelators/acid leach metals out	Fast, high efficiency	Destroys soil structure, secondary pollution risk	Moderately polluted farmland	Pb, Cd removal 50%–90%
–	Stabilization/solidification	Add lime, phosphate, biochar to immobilize	Low cost, large area applicable	Does not remove, only transforms	Paddy, dryland common	Cd uptake reduction 30%–50%
**Biological**	Phytoremediation	Hyperaccumulators absorb and remove via harvest	Low cost, eco-friendly	Long cycle, climate limited	Lightly polluted soil for Cd, As, Zn	As accumulation factor >10
–	Microbial remediation	Microbes transform/precipitate/immobilize	No secondary pollution, eco-friendly	Influenced by environment	Cr, Hg polluted soil	Cr(VI) conversion >80%
**Agronomic regulation**	Organic fertilizer/straw return	Increase OM adsorption	Improves soil quality, low cost	Limited effect	Farmland common	Rice grain Cd reduction 30%–50%
**Latest tech**	Nanomaterial remediation	Use nanomaterials to adsorb/reduce metals	Significant effect, fast reaction	High cost, potential eco-risk	Cr, As, Pb polluted soil	Cd available form reduction >70%
–	Intelligent remediation	AI prediction + precise dosing	High efficiency, real-time monitoring	High tech cost, early application	Site remediation and farmland monitoring	Remediation prediction accuracy >90%

Note, the table and all related contents were compiled by the author.

For co-contaminated sites, integrated remediation strategies must address the distinct geochemical behaviors of multiple metals. In Cd-As co-contaminated paddy soils, water management presents a trade-off: flooding reduces Cd availability but increases As mobility, while intermittent flooding decreases As but may enhance Cd uptake. A combined approach of intermittent flooding coupled with iron-based amendments (e.g., iron sulfate, biochar-supported iron oxides) has been shown to simultaneously reduce grain Cd and As by 30%–50%, as iron oxides immobilize Cd through adsorption and As through co-precipitation. For Pb-Cd co-contaminated upland soils, phosphate amendments (e.g., apatite, phosphate rock, etc.) can immobilize Pb via pyromorphite formation while also reducing Cd bioavailability through pH-induced adsorption. Additionally, the use of mixed amendments (lime + biochar + zeolite) has demonstrated synergistic effects in multi-metal immobilization, achieving >60% reduction in available Pb, Cd, and Cu in field trials. Plant selection also plays a critical role; breeding or screening for low-accumulation cultivars that exhibit co-tolerance to multiple metals (e.g., Cd-As low-accumulation rice varieties) offers a sustainable, cost-effective strategy for safe production in co-contaminated regions. These examples underscore that effective management of combined pollution requires moving beyond single-metal approaches toward integrated, site-specific strategies that account for metal-metal interactions and their differential responses to environmental conditions. A comparison of different remediation technologies for metal-contaminated soil is provided in Table 5.

#### Agronomic measures regulation

Improving farming systems and fertilization management can reduce heavy metal bioavailability to some extent. Applying organic fertilizer, straw return increases soil organic matter, enhancing heavy metal complexation and fixation; applying alkaline materials like lime, dolomite increases soil pH, causing Cd, Pb, etc., to form hydroxide precipitates, reducing crop uptake. Experiments prove applying 1,500 kg/ha lime in Cd-polluted paddy reduces rice grain Cd content by 30%–50%.^143^ Additionally, practices like crop rotation and deep plowing can change heavy metal distribution in soil profiles, mitigating agricultural product pollution risk.

#### Latest technological advances

##### Nanomaterial remediation

Nanomaterials show unique advantages in heavy metal remediation due to large specific surface area, strong surface activity, and fast reaction rates. Zero-valent iron nanoparticles (nZVI) can reduce Cr(VI) to Cr(III) precipitate, and form precipitates or adsorb As, Cd, reducing mobility and toxicity.^144^ Additionally, nanomaterials like nano iron oxide, nano hydroxyapatite, carbon nanotubes can immobilize heavy metals via adsorption, complexation, or ion exchange.^145^ Studies show applying 1% nZVI can reduce exchangeable Cd in soil by ∼70%. However, nanomaterials have issues like high cost, easy aggregation, potential ecological risks, requiring composite use with other materials (e.g., nZVI-biochar composites) to improve stability and environmental friendliness.

##### Green low-carbon remediation materials

To address secondary pollution from traditional chemical remediation, researchers propose using green low-carbon materials for heavy metal stabilization. Common materials include biochar, mineral-based amendments (zeolite, bentonite, etc.), modified agricultural by-products (e.g., rice husk ash, bone meal, etc.). Biochar, due to its porous structure and abundant functional groups, significantly improves soil buffer capacity and heavy metal adsorption.^146^ For example, applying rice husk biochar (20 t/ha) in Cd-polluted soil reduces crop Cd content over 40%. Green remediation materials are widely sourced, low-cost, and have carbon sequestration/emission reduction effects, aligning with sustainable development.

##### Intelligent and precision remediation

With AI, big data, and sensor technology development, intelligent remediation is becoming a research focus. Deploying soil sensors enables real-time monitoring of soil heavy metal content, pH, Eh, etc., combined with UAV remote sensing and GIS for refined survey and dynamic monitoring of polluted sites.^147^ During remediation, AI algorithms can optimize amendment dosage, repair time, and environmental conditions, improving efficiency and reducing cost. For example, machine learning prediction models can predict post-remediation heavy metal bioavailability with >90% accuracy. Future intelligent remediation may integrate with blockchain for transparent supervision of the entire process.

##### Combined and coupled remediation

Single remediation technologies often have limitations, hence researchers propose “multi-technology joint” models. For instance, plant-microbe combined remediation significantly improves efficiency^148^; electrokinetic-chemical washing coupling accelerates heavy metal migration in low-permeability soil. Cases show when electrokinetic remediation couples with EDDS washing, soil Ni and Cu removal rates increase 30%–40%.^149^ Combined remediation overcomes single-technology shortcomings and achieves “rapid remediation + long-term stability,” an important future direction.

### Comprehensive management and policy support

In practice, single remediation measures often fail to achieve ideal results. A full-chain management approach of “source control-pollution blocking-ecological restoration-risk management” should be comprehensively adopted.

International case studies demonstrate the feasibility of integrated remediation strategies across diverse contexts:

United States (EPA Superfund sites), multiple projects exemplify successful ecological revitalization. At the Bunker Hill Superfund site (Idaho), biosolids and compost caps restored wetland ecosystem function on highly contaminated Pb-Zn tailings.^150^ At the Palmerton Zinc Pile site (Pennsylvania), soil amendments and revegetation restored 70% of vegetative cover on over 2,000 acres deforested by smelting emissions. At the California Gulch site (Colorado), lime and biosolids applications revitalized the upper Arkansas river, enabling trout populations to thrive where they could not survive for decades.

Spain (La Soterraña, Asturias), a former Hg mine with >80,000 tonnes of waste and As concentrations reaching 40 mg/L in leachates is being remediated using a hybrid nature-based system combining adsorptive filtration, organic amendments, and phytoremediation, demonstrating a scalable, cost-effective approach for legacy mining sites where conventional treatment is unfeasible.^151^

Italy (Sardinia), at the Ingurtosu former mining site, researchers are testing bioaugmentation using 11 native bacterial strains isolated from mining waste. These bacteria promote plant growth, improve soil biodiversity, and help immobilize heavy metals (particularly Pb and Zn), contributing to soil stabilization without degrading metals—offering a sustainable, replicable model.^152^

These international examples share common success factors: nature-based solutions (phytoremediation, bioaugmentation, etc.) that minimize energy input; adaptive management tailored to site-specific conditions; multi-stakeholder partnerships linking industry, academia, and government; and long-term monitoring to ensure sustained effectiveness. These lessons can inform remediation strategies globally, including in China, where ongoing pilot projects—such as those combining chemical stabilization with agronomic regulation—have demonstrated that region-specific, integrated approaches can achieve both short-term risk reduction and long-term agricultural sustainability.

### Medical prevention and treatment pathways

#### Cut off exposure sources

Heavy metal toxicity in humans often manifests as chronic latent damage.^153^ The primary medical prevention step is cutting off exposure sources, including improving drinking water sources, controlling food safety, strengthening occupational protection, and environmental remediation. For example, in South Asia, over 40 million people chronically drink As-contaminated groundwater; switching to low-As water sources significantly reduced local arsenism incidence.^154^ In Pb exposure control, the US phased out leaded gasoline and lead-based paints, reducing average child blood Pb levels by over 80% in 20 years, significantly lowering neurotoxicity risk.

#### Pharmaceutical intervention

Drug intervention plays a key role in treating heavy metal poisoning. Chelating agents are most commonly used, promoting excretion by forming stable complexes with metal ions, e.g., EDTA, DMSA, and DMPS widely used for Pb, Hg, and As poisoning.^155^ Clinical studies show children receiving DMSA treatment can reduce blood Pb levels by 30%–50% within weeks. In Wilson’s disease patients, penicillamine and trientine effectively promote Cu excretion, combined with Zn preparations inhibiting intestinal Cu absorption,^156^ reducing serum Cu levels over 60%. Chelation therapy is not harmless, may cause electrolyte imbalance or liver/kidney damage, requiring strict monitoring.

#### Nutritional intervention

Nutritional intervention is another important measure in medical prevention. Multiple studies show Ca, Fe, and Zn supplementation can significantly reduce intestinal absorption of Cd and Pb^157^; folic acid and selenium help promote As methylation metabolism and excretion, reducing its CR.^158^ Pilot studies in Japan and Bangladesh show folic acid supplementation increases methylated As proportion in urine by ∼15%, demonstrating the potential of nutritional strategies against arsenism. For Hg-exposed populations, selenium supplementation is proven to reduce toxicity by forming Hg-Se complexes^103^; some studies find fish consumers increasing dietary selenium show significantly reduced blood Hg-related neurodamage indicators.

#### Supportive and symptomatic treatment

Supportive treatment is also important in alleviating heavy metal damage. For example, Cr poisoning patients can use antioxidants (Vit C, glutathione) to reduce DNA oxidative damage; Cu/Fe deficiency anemia from Zn excess can be corrected by supplementing Cu and Fe. Recently, research focuses on adjuvant therapy based on antioxidants and natural products, e.g., N-acetylcysteine (NAC) and polyphenols show application potential in protecting liver/kidney function and alleviating heavy metal toxicity due to antioxidant and anti-inflammatory properties.^159^

Overall, medical prevention of heavy metal toxicity emphasizes a comprehensive strategy of “exposure blocking-drug-promoted excretion-nutritional regulation-supportive treatment.” Future directions include developing more efficient, less toxic chelators, exploring precise nutritional intervention strategies, and identifying high-risk populations using genomics and metabolomics for more targeted health protection.

## Conclusion and outlook

In recent years, with the accelerated development of industrialization, urbanization, and agricultural intensification, heavy metal pollution has become a significant environmental issue threatening ecosystem security and public health. This study systematically reviews crop-mediated heavy metal exposure pathways and their harm mechanisms to human health from an environmental health perspective. Results show that heavy metal pollution sources are complex, including high-concentration emissions from industrial activities such as mining, smelting, electroplating, and chemical production; chronic inputs from agricultural practices like phosphate fertilizer application, sewage irrigation, and livestock manure; combined with the cumulative effects from domestic sources and historical pesticide legacy. In this context, soil becomes the primary sink and reservoir for heavy metals, while crops become the key link transferring pollution to humans via root uptake, transmembrane transport, and internal distribution. Numerous studies and cases demonstrate that staple crops like rice, wheat, corn, and leafy and root vegetables exhibit significant accumulation capacity, not only reducing agricultural product yield and quality but also inducing major health risks like chronic poisoning, nervous system damage, kidney disease, and cancer through long-term dietary exposure. Clearly, crop-mediated heavy metal exposure has become a core hidden danger constraining food security and public health. Constructing a full-chain prevention and control system of “source governance-farmland regulation-food safety-public health” is imperative.

Building upon this synthesis, we propose the following concrete and actionable recommendations for future research, policy, and practice to effectively mitigate crop-mediated heavy metal risks:

Develop predictive models for bioavailability: future research should prioritize the development of predictive models that quantify how key environmental factors—such as soil acidification rates, changing organic matter content, and specific irrigation regimes (e.g., alternate wetting and drying)—interact to alter the bioavailability of different heavy metals. This requires moving beyond descriptive studies to establishing quantitative relationships that can inform field management.

Establish dynamic food safety standards: national and international bodies (e.g., Codex Alimentarius) should be urged to move toward dynamic, risk-based food safety standards. This includes regularly updating maximum contaminant levels based on new toxicological evidence and dietary exposure patterns, and clearly explaining the rationale for any revisions to stakeholders.

Develop an integrated “Soil-Food-Health” monitoring system: policymakers should invest in creating a nationally coordinated, cross-departmental monitoring platform that integrates soil quality data, crop contaminant surveillance, and human biomonitoring (e.g., blood Pb levels). This AI-assisted system should be designed to provide early warnings of emerging risks and enable targeted interventions in identified hotspots.

Launch targeted dietary guidance campaigns: public health agencies should develop and disseminate targeted dietary guidelines for populations living in known heavy metal pollution hotspots. These guidelines should provide practical advice, such as which locally grown vegetables to consume in moderation and food preparation methods that can reduce heavy metal content (e.g., thorough washing, specific cooking techniques, etc.).

In conclusion, effectively addressing the challenge of crop-mediated heavy metal exposure demands a concerted, multi-disciplinary effort. By pursuing these targeted research avenues, implementing robust and adaptive policies, and engaging communities through education, we can build a resilient defense system. This integrated approach will be essential for safeguarding human health, ensuring sustainable food production, and achieving the broader goals of global environmental sustainability and the “Healthy China” initiative.

### Availability of data and materials

The datasets analyzed during the current study are available from the corresponding author on reasonable request.

## Acknowledgments

The authors would like to thank all the anonymous referees for their constructive comments and suggestions. Funding, the authors are grateful for the support from the 10.13039/501100007129Natural Science Foundation of Shandong Province (ZR2022QC193), 10.13039/501100001809National Natural Science Foundation of China (32370363) and 10.13039/501100007129Natural Science Foundation of Shandong Province (ZR2024QD059).

## Declaration of interests

The authors declare no competing interests.

## References

[1] Yu J., Chen Z., Gao W., He S., Xiao D., Fan W., Huo M., Nugroho W.A. (2025). Global trends and prospects in research on heavy metal pollution at contaminated sites. J. Environ. Manag..

[2] Xu W., Jin Y., Zeng G. (2024). Introduction of heavy metals contamination in the water and soil: a review on source, toxicity and remediation methods. Green Chem. Lett. Rev..

[3] Jomova K., Alomar S.Y., Nepovimova E., Kuca K., Valko M. (2025). Heavy metals: toxicity and human health effects. Arch. Toxicol..

[4] Hou D., Jia X., Wang L., McGrath S.P., Zhu Y.-G., Hu Q., Zhao F.-J., Bank M.S., O’Connor D., Nriagu J. (2025). Global soil pollution by toxic metals threatens agriculture and human health. Science.

[5] Shi J., Zhao D., Ren F., Huang L. (2023). Spatiotemporal variation of soil heavy metals in China: The pollution status and risk assessment. Sci. Total Environ..

[6] Grandjean P., Satoh H., Murata K., Eto K. (2010). Adverse effects of methylmercury: environmental health research implications. Environ. Health Perspect..

[7] Hernandez-Soriano M.C., Jimenez-Lopez J.C. (2012). Effects of soil water content and organic matter addition on the speciation and bioavailability of heavy metals. Sci. Total Environ..

[8] Ijaz S., Iqbal J., Abbasi B.A., Tufail A., Ullah Z., Yaseen T., Ali I., Uddin S., Iqbal R. (2024). Biochar-assisted remediation of contaminated soils under changing climate.

[9] Yuan Z., Luo T., Liu X., Hua H., Zhuang Y., Zhang X., Zhang L., Zhang Y., Xu W., Ren J. (2019). Tracing anthropogenic cadmium emissions: From sources to pollution. Sci. Total Environ..

[10] Chen H., Yang X., Wang P., Wang Z., Li M., Zhao F.-J. (2018). Dietary cadmium intake from rice and vegetables and potential health risk: A case study in Xiangtan, southern China. Sci. Total Environ..

[11] Rasin P., V A.A., Basheer S.M., Haribabu J., Santibanez J.F., Garrote C.A., Arulraj A., Mangalaraja R.V. (2025). Exposure to cadmium and its impacts on human health: A short review. J. Hazard. Mater. Adv..

[12] Xiao R., Guo D., Ali A., Mi S., Liu T., Ren C., Li R., Zhang Z. (2019). Accumulation, ecological-health risks assessment, and source apportionment of heavy metals in paddy soils: A case study in Hanzhong, Shaanxi, China. Environ. Pollut..

[13] Rizwan M., Ali S., Adrees M., Ibrahim M., Tsang D.C.W., Zia-ur-Rehman M., Zahir Z.A., Rinklebe J., Tack F.M.G., Ok Y.S. (2017). A critical review on effects, tolerance mechanisms and management of cadmium in vegetables. Chemosphere.

[14] Obeng-Gyasi E. (2019). Sources of lead exposure in various countries. Rev. Environ. Health.

[15] Mitra P., Sharma S., Purohit P., Sharma P. (2017). Clinical and molecular aspects of lead toxicity: An update. Crit. Rev. Clin. Lab Sci..

[16] Souri M.K., Hatamian M., Tesfamariam T. (2019). Plant growth stage influences heavy metal accumulation in leafy vegetables of garden cress and sweet basil. Chem. Biol. Technol. Agric..

[17] Pan S., Lin L., Zeng F., Zhang J., Dong G., Yang B., Jing Y., Chen S., Zhang G., Yu Z. (2018). Effects of lead, cadmium, arsenic, and mercury co-exposure on children's intelligence quotient in an industrialized area of southern China. Environ. Pollut..

[18] Du H., Guo P., Wang T., Ma M., Wang D. (2021). Significant bioaccumulation and biotransformation of methyl mercury by organisms in rice paddy ecosystems: A potential health risk to humans. Environ. Pollut..

[19] Farina M., Rocha J.B.T., Aschner M. (2011). Mechanisms of methylmercury-induced neurotoxicity: evidence from experimental studies. Life Sci..

[20] Guo P., Du H., Wang D., Ma M. (2021). Effects of mercury stress on methylmercury production in rice rhizosphere, methylmercury uptake in rice and physiological changes of leaves. Sci. Total Environ..

[21] Netto B.B., da Silva E.P., de Aguiar da Costa M., de Rezende V.L., Bolan S.J., Ceretta L.B., Aschner M., Dominguini D., Gonçalves C.L. (2024). Critical period of exposure to mercury and the diagnostic of autism spectrum disorder: A systematic review. J. Neurochem..

[22] Paltseva A., Cheng Z., Deeb M., Groffman P.M., Shaw R.K., Maddaloni M. (2018). Accumulation of arsenic and lead in garden-grown vegetables: Factors and mitigation strategies. Sci. Total Environ..

[23] Wei X., Zhou Y., Tsang D.C.W., Song L., Zhang C., Yin M., Liu J., Xiao T., Zhang G., Wang J. (2020). Hyperaccumulation and transport mechanism of thallium and arsenic in brake ferns (Pteris vittata L.): A case study from mining area. J. Hazard Mater..

[24] Wan Y., Huang Q., Camara A.Y., Wang Q., Li H. (2019). Water management impacts on the solubility of Cd, Pb, As, and Cr and their uptake by rice in two contaminated paddy soils. Chemosphere.

[25] Nurchi V.M., Buha Djordjevic A., Crisponi G., Alexander J., Bjørklund G., Aaseth J. (2020). Arsenic Toxicity: Molecular Targets and Therapeutic Agents. Biomolecules.

[26] Wang Z., Guo H.-m., Liu H.-y., Zhang W.-m. (2023). Source, migration, distribution, toxicological effects and remediation technologies of arsenic in groundwater in China. China Geol..

[27] Sharma P., Singh S.P., Parakh S.K., Tong Y.W. (2022). Health hazards of hexavalent chromium (Cr (VI)) and its microbial reduction. Bioengineered.

[28] Singh V., Singh N., Verma M., Kamal R., Tiwari R., Sanjay Chivate M., Rai S.N., Kumar A., Singh A., Singh M.P. (2022). Hexavalent-chromium-induced oxidative stress and the protective role of antioxidants against cellular toxicity. Antioxidants.

[29] López-Luna J., González-Chávez M.C., Esparza-García F.J., Rodríguez-Vázquez R. (2009). Toxicity assessment of soil amended with tannery sludge, trivalent chromium and hexavalent chromium, using wheat, oat and sorghum plants. J. Hazard Mater..

[30] Singh V., Singh N., Verma M., Kamal R., Tiwari R., Sanjay Chivate M., Rai S.N., Kumar A., Singh A., Singh M.P. (2022). Hexavalent-Chromium-Induced Oxidative Stress and the Protective Role of Antioxidants against Cellular Toxicity. Antioxidants.

[31] Kapoor R.T., Bani Mfarrej M.F., Alam P., Rinklebe J., Ahmad P. (2022). Accumulation of chromium in plants and its repercussion in animals and humans. Environ. Pollut..

[32] Du B., Zhou J., Lu B., Zhang C., Li D., Zhou J., Jiao S., Zhao K., Zhang H. (2020). Environmental and human health risks from cadmium exposure near an active lead-zinc mine and a copper smelter, China. Sci. Total Environ..

[33] Zhao H., Wu L., Chai T., Zhang Y., Tan J., Ma S. (2012). The effects of copper, manganese and zinc on plant growth and elemental accumulation in the manganese-hyperaccumulator Phytolacca americana. J. Plant Physiol..

[34] Sun Z., Shao Y., Yan K., Yao T., Liu L., Sun F., Wu J., Huang Y. (2023). The Link between Trace Metal Elements and Glucose Metabolism: Evidence from Zinc, Copper, Iron, and Manganese-Mediated Metabolic Regulation. Metabolites.

[35] Silverberg N.B., Pelletier J.L., Jacob S.E., Schneider L.C., Cohen B., Horii K.A., Kristal L., Maguiness S.M., Tollefson M.M., Weinstein M.G. (2020). Nickel Allergic Contact Dermatitis: Identification, Treatment, and Prevention. Pediatrics.

[36] Kalungi P., Yao Z., Huang H. (2024). Aspects of nickel, cobalt and lithium, the three key elements for Li-ion batteries: an overview on resources, demands, and production. Materials.

[37] Liu T., Li F., Jin Z., Yang Y. (2018). Acidic leaching of potentially toxic metals cadmium, cobalt, chromium, copper, nickel, lead, and zinc from two Zn smelting slag materials incubated in an acidic soil. Environ. Pollut..

[38] Ma T., Luo H., Sun J., Dang Z., Lu G. (2024). The effect of heavy precipitation on the leaching of heavy metals from tropical coastal legacy tailings. Waste Manag..

[39] Liu J., Qiao S., Chen H., Zhao S., Li C., Wu Y., Li D., Li L. (2024). Multiple pathway exposure risks and driving factors of heavy metals in soil-crop system in a Pb/Zn smelting city, China. J. Clean. Prod..

[40] Wu X., Su N., Yue X., Fang B., Zou J., Chen Y., Shen Z., Cui J. (2021). IRT1 and ZIP2 were involved in exogenous hydrogen-rich water-reduced cadmium accumulation in Brassica chinensis and Arabidopsis thaliana. J. Hazard Mater..

[41] Ma J.F., Yamaji N., Mitani N., Xu X.-Y., Su Y.-H., McGrath S.P., Zhao F.-J. (2008). Transporters of arsenite in rice and their role in arsenic accumulation in rice grain. Proc. Natl. Acad. Sci. USA.

[42] Li Z. (2022). Modeling plant uptake of organic contaminants by root vegetables: The role of diffusion, xylem, and phloem uptake routes. J. Hazard Mater..

[43] Schipper L.A., Sparling G.P., Fisk L.M., Dodd M.B., Power I.L., Littler R.A. (2011). Rates of accumulation of cadmium and uranium in a New Zealand hill farm soil as a result of long-term use of phosphate fertilizer. Agric. Ecosyst. Environ..

[44] Alengebawy A., Abdelkhalek S.T., Qureshi S.R., Wang M.-Q. (2021). Heavy Metals and Pesticides Toxicity in Agricultural Soil and Plants: Ecological Risks and Human Health Implications. Toxics.

[45] Meng W., Wang Z., Hu B., Wang Z., Li H., Goodman R.C. (2016). Heavy metals in soil and plants after long-term sewage irrigation at Tianjin China: A case study assessment. Agric. Water Manag..

[46] Peng S., Zhang H., Song D., Chen H., Lin X., Wang Y., Ji L. (2022). Distribution of antibiotic, heavy metals and antibiotic resistance genes in livestock and poultry feces from different scale of farms in Ningxia, China. J. Hazard Mater..

[47] Ulrich A.E. (2019). Cadmium governance in Europe's phosphate fertilizers: Not so fast?. Sci. Total Environ..

[48] Shi T., Ma J., Wu X., Ju T., Lin X., Zhang Y., Li X., Gong Y., Hou H., Zhao L., Wu F. (2018). Inventories of heavy metal inputs and outputs to and from agricultural soils: A review. Ecotoxicol. Environ. Saf..

[49] Yang Q., Li Z., Lu X., Duan Q., Huang L., Bi J. (2018). A review of soil heavy metal pollution from industrial and agricultural regions in China: Pollution and risk assessment. Sci. Total Environ..

[50] Yang S., Feng W., Wang S., Chen L., Zheng X., Li X., Zhou D. (2021). Farmland heavy metals can migrate to deep soil at a regional scale: A case study on a wastewater-irrigated area in China. Environ. Pollut..

[51] Dutta D., Goel S. (2021). Understanding the gap between formal and informal e-waste recycling facilities in India. Waste Manag..

[52] Wang M., Jiang D., Ding D., Deng S., Kong L., Wei J., Xia F., Li M., Long T. (2023). Spatiotemporal characteristics and dynamic risk assessment of a multi-solvents abandoned pesticide-contaminated site with a long history, in China. J. Environ. Manag..

[53] Eboigbe E.O., Veerasamy N., Odukoya A.M., Anene N.C., Sonke J.E., Sagisaka Méndez S., McLagan D.S. (2025). Mercury contamination in staple crops impacted by artisanal and small-scale gold mining (ASGM): stable Hg isotopes demonstrate dominance of atmospheric uptake pathway for Hg in crops. Biogeosciences.

[54] Xu S., Yun M., Wang Y., Liu K., Wu A., Li S., Su Y., Wang S., Kang H. (2025). Heavy Metal Contamination and Risk Assessment in Soil–Wheat/Corn Systems near Metal Mining Areas in Northwestern China. Biology.

[55] Thakur S., Singh L., Wahid Z.A., Siddiqui M.F., Atnaw S.M., Din M.F.M. (2016). Plant-driven removal of heavy metals from soil: uptake, translocation, tolerance mechanism, challenges, and future perspectives. Environ. Monit. Assess..

[56] Marr K., Fyles H., Hendershot W. (1999). Trace metals in Montreal urban soils and the leaves of Taraxacum officinale. Can. J. Soil Sci..

[57] Mapanda F., Mangwayana E.N., Nyamangara J., Giller K.E. (2005). The effect of long-term irrigation using wastewater on heavy metal contents of soils under vegetables in Harare, Zimbabwe. Agric. Ecosyst. Environ..

[58] Wu X., Lin Q., Li G., Guo C., Li L., Wang J. (2024). Evaluating water management efficiency in regulating cadmium and arsenic accumulation in rice in typical japonica paddy soils at varied pH levels. Agriculture.

[59] Zheng X., Lin H., Du D., Li G., Alam O., Cheng Z., Liu X., Jiang S., Li J. (2024). Remediation of heavy metals polluted soil environment: A critical review on biological approaches. Ecotoxicol. Environ. Saf..

[60] Podar D., Maathuis F.J.M. (2022). The role of roots and rhizosphere in providing tolerance to toxic metals and metalloids. Plant Cell Environ..

[61] Yu Y., Zheng H., Chen Q., Fei J., Sun H., Ding Z. (2025). Unraveling the pathways of heavy metal accumulation in rice: roles of roots, stems, and soil pH. Ecotoxicol. Environ. Saf..

[62] Walne C.H., Reddy K.R. (2022). Temperature effects on the shoot and root growth, development, and biomass accumulation of corn (Zea mays L.). Agriculture.

[63] Zhang J., Qian Y., Chen Z., Amee M., Niu H., Du D., Yao J., Chen K., Chen L., Sun J. (2020). Lead-induced oxidative stress triggers root cell wall remodeling and increases lead absorption through esterification of cell wall polysaccharide. J. Hazard Mater..

[64] Pastor K., Nastić N., Ilić M., Skendi A., Stefanou S., Ačanski M., Rocha J.M., Papageorgiou M. (2024). A screening study of elemental composition in legume (Fabaceae sp.) cultivar from Serbia: Nutrient accumulation and risk assessment. J. Food Compos. Anal..

[65] Bi C., Zhou Y., Chen Z., Jia J., Bao X. (2018). Heavy metals and lead isotopes in soils, road dust and leafy vegetables and health risks via vegetable consumption in the industrial areas of Shanghai, China. Sci. Total Environ..

[66] Ma J.F., Shen R.F., Shao J.F. (2021). Transport of cadmium from soil to grain in cereal crops: A review. Pedosphere.

[67] Mubeen S., Ni W., He C., Yang Z. (2023). Agricultural strategies to reduce cadmium accumulation in crops for food safety. Agriculture.

[68] Zhang W., Guan M., Chen M., Lin X., Xu P., Cao Z. (2024). Mutation of OsNRAMP5 reduces cadmium xylem and phloem transport in rice plants and its physiological mechanism. Environ. Pollut..

[69] Xie L., Hao P., Cheng Y., Ahmed I.M., Cao F. (2018). Effect of combined application of lead, cadmium, chromium and copper on grain, leaf and stem heavy metal contents at different growth stages in rice. Ecotoxicol. Environ. Saf..

[70] Kosakivska I.V., Babenko L.M., Romanenko K.O., Korotka I.Y., Potters G. (2021). Molecular mechanisms of plant adaptive responses to heavy metals stress. Cell Biol. Int..

[71] Goni M.A., Hosen L., Khan A.S., Abdullah-Al-Mamun M., Khatun M.J., Siddiquee T. (2025). Elevated uptake and translocation patterns of heavy metals in different food plants parts and their impacts on human health. Biol. Trace Elem. Res..

[72] Domínguez Carrasco M.D., Salo T., Keskinen R., Suomi J. (2025). Comparison of the Effects of Bio-Based and Mineral Fertiliser Use on Heavy Metals Dietary Exposure in Six European Countries. Agric. Food Sci..

[73] Cao Y., Ma C., Chen H., Zhang J., White J.C., Chen G., Xing B. (2020). Xylem-based long-distance transport and phloem remobilization of copper in Salix integra Thunb. J. Hazard Mater..

[74] Hassan H., Elaksher S.H., Shabala S., Ouyang B. (2024). Cadmium uptake and detoxification in tomato plants: Revealing promising targets for genetic improvement. Plant Physiol. Biochem..

[75] Ye X.X., Wang G.Z., Zhang Y.X., Zhao H.J. (2018). Hydroxyapatite nanoparticles in root cells: reducing the mobility and toxicity of Pb in rice. Environ. Sci. Nano.

[76] Meng Y.T., Zhang X.L., Wu Q., Shen R.F., Zhu X.F. (2022). Transcription factor ANAC004 enhances Cd tolerance in Arabidopsis thaliana by regulating cell wall fixation, translocation and vacuolar detoxification of Cd, ABA accumulation and antioxidant capacity. J. Hazard Mater..

[77] Rahman M.M., Sarkar M.I.U., Anderson Z., Hosain M.T., Sharma A., Asadi S., Naidu R. (2025). Multivariate and predictive modelling of arsenic and cadmium in rice: Influence of origin, grain type, and processing. Environ. Pollut..

[78] Han R., Wang Z., Wang S., Sun G., Xiao Z., Hao Y., Nriagu J., Teng H.H., Li G. (2023). A combined strategy to mitigate the accumulation of arsenic and cadmium in rice (Oryza sativa L.). Sci. Total Environ..

[79] Singh S., Zacharias M., Kalpana S., Mishra S. (2012). Heavy metals accumulation and distribution pattern in different vegetable crops. J. Environ. Chem. Ecotoxicol..

[80] Zhang X., Yang M., Yang H., Pian R., Wang J., Wu A.-M. (2024). The uptake, transfer, and detoxification of cadmium in plants and its exogenous effects. Cells.

[81] Vasilachi I.C., Stoleru V., Gavrilescu M. (2023). Analysis of Heavy Metal Impacts on Cereal Crop Growth and Development in Contaminated Soils. Agriculture.

[82] Valdez C.E., Smith Q.A., Nechay M.R., Alexandrova A.N. (2014). Mysteries of metals in metalloenzymes. Acc. Chem. Res..

[83] Huihui Z., Xin L., Zisong X., Yue W., Zhiyuan T., Meijun A., Yuehui Z., Wenxu Z., Nan X., Guangyu S. (2020). Toxic effects of heavy metals Pb and Cd on mulberry (Morus alba L.) seedling leaves: Photosynthetic function and reactive oxygen species (ROS) metabolism responses. Ecotoxicol. Environ. Saf..

[84] Huang L., Li W.C., Tam N.F.Y., Ye Z. (2019). Effects of root morphology and anatomy on cadmium uptake and translocation in rice (Oryza sativa L.). JES (J. Environ. Sci.).

[85] Xia W., Ghouri F., Zhong M., Bukhari S.A.H., Ali S., Shahid M.Q. (2024). Rice and heavy metals: A review of cadmium impact and potential remediation techniques. Sci. Total Environ..

[86] Tamás L., Mistrík I., Zelinová V. (2017). Heavy metal-induced reactive oxygen species and cell death in barley root tip. Environ. Exp. Bot..

[87] Liu J., Gai L., Zong H. (2021). Foliage application of chitosan alleviates the adverse effects of cadmium stress in wheat seedlings (Triticum aestivum L.). Plant Physiol. Biochem..

[88] Huang H., Zheng X., Tao S., Ma S., Feng S., Li Y., Liang K., Li G., Lei J., Liu H., Xu H. (2025). Adaptive mechanisms of moss (Campylopus schmidii) to Cr(Ⅵ) stress in pyrite-mining polluted ecosystems: Integrating biochemical, structural, and molecular perspectives. Environ. Pollut..

[89] Nkongolo K., Michael P. (2024). Reduced representation bisulfite sequencing (RRBS) analysis reveals variation in distribution and levels of DNA methylation in white birch (Betula papyrifera) exposed to nickel. Genome.

[90] Lu L., Chen X., Chen J., Zhang Z., Zhang Z., Sun Y., Wang Y., Xie S., Ma Y., Song Y., Zeng R. (2024). MicroRNA-encoded regulatory peptides modulate cadmium tolerance and accumulation in rice. Plant Cell Environ..

[91] Ding Y., Gong S., Wang Y., Wang F., Bao H., Sun J., Cai C., Yi K., Chen Z., Zhu C. (2018). MicroRNA166 modulates cadmium tolerance and accumulation in rice. Plant Physiol..

[92] Ran T., Cao G., Xiao L., Li Y., Xia R., Zhao X., Qin Y., Wu P., Tian S. (2024). Effects of cadmium stress on the growth and physiological characteristics of sweet potato. BMC Plant Biol..

[93] Jawad Hassan M., Ali Raza M., Ur Rehman S., Ansar M., Gitari H., Khan I., Wajid M., Ahmed M., Abbas Shah G., Peng Y., Li Z. (2020). Effect of Cadmium Toxicity on Growth, Oxidative Damage, Antioxidant Defense System and Cadmium Accumulation in Two Sorghum Cultivars. Plants.

[94] Zhang X., Chen J., Wang W., Zhu L. (2024). Photosynthetic mechanisms of carbon fixation reduction in rice by cadmium and polycyclic aromatic hydrocarbons. Environ. Pollut..

[95] Smith A.T., Barupala D., Stemmler T.L., Rosenzweig A.C. (2015). A new metal binding domain involved in cadmium, cobalt and zinc transport. Nat. Chem. Biol..

[96] Bouillaud F. (2023). Inhibition of succinate dehydrogenase by pesticides (SDHIs) and energy metabolism. Int. J. Mol. Sci..

[97] Beniwal R., Yadav R., Ramakrishna W. (2023). Multifarious effects of arsenic on plants and strategies for mitigation. Agriculture.

[98] Meaza I., Williams A.R., Wise S.S., Lu H., Wise J.P. (2024). Carcinogenic mechanisms of hexavalent chromium: From DNA breaks to chromosome instability and neoplastic transformation. Curr. Environ. Health Rep..

[99] Zhang Y., Yang C., Liu S., Xie Z., Chang H., Wu T. (2024). Phytohormones-mediated strategies for mitigation of heavy metals toxicity in plants focused on sustainable production. Plant Cell Rep..

[100] Zažímalová E., Křeček P., Skůpa P., Hoyerová K., Petrášek J. (2007). Polar transport of the plant hormone auxin–the role of PIN-FORMED (PIN) proteins. Cell. Mol. Life Sci..

[101] Wang H.-Q., Xuan W., Huang X.-Y., Mao C., Zhao F.-J. (2021). Cadmium inhibits lateral root emergence in rice by disrupting OsPIN-mediated auxin distribution and the protective effect of OsHMA3. Plant Cell Physiol..

[102] Niekerk L.A., Gokul A., Basson G., Badiwe M., Nkomo M., Klein A., Keyster M. (2024). Heavy metal stress and mitogen activated kinase transcription factors in plants: Exploring heavy metal-ROS influences on plant signalling pathways. Plant Cell Environ..

[103] Yang D.-Y., Chen Y.-W., Gunn J.M., Belzile N. (2008). Selenium and mercury in organisms: Interactions and mechanisms. Environ. Rev..

[104] Sharma S.S., Kumar V., Dietz K.-J. (2021). Emerging trends in metalloid-dependent signaling in plants. Trends Plant Sci..

[105] Zhang H., Lu L. (2024). Transcription factors involved in plant responses to cadmium-induced oxidative stress. Front. Plant Sci..

[106] Kaur R., Das S., Bansal S., Singh G., Sardar S., Dhar H., Ram H. (2021). Heavy metal stress in rice: Uptake, transport, signaling, and tolerance mechanisms. Physiol. Plantarum.

[107] Sánchez-Corona C.G., Gonzalez-Avila L.U., Hernández-Cortez C., Rojas-Vargas J., Castro-Escarpulli G., Castelán-Sánchez H.G. (2025). Impact of heavy metal and resistance genes on antimicrobial resistance: ecological and public health implications. Genes.

[108] Uddin M.M., Zakeel M.C.M., Zavahir J.S., Marikar F.M.M.T., Jahan I. (2021). Heavy metal accumulation in rice and aquatic plants used as human food: A general review. Toxics.

[109] Zeng F., Ali S., Zhang H., Ouyang Y., Qiu B., Wu F., Zhang G. (2011). The influence of pH and organic matter content in paddy soil on heavy metal availability and their uptake by rice plants. Environ. Pollut..

[110] Mitra S., Chakraborty A.J., Tareq A.M., Emran T.B., Nainu F., Khusro A., Idris A.M., Khandaker M.U., Osman H., Alhumaydhi F.A., Simal-Gandara J. (2022). Impact of heavy metals on the environment and human health: Novel therapeutic insights to counter the toxicity. J. King Saud Univ. Sci..

[111] Román-Ochoa Y., Choque Delgado G.T., Tejada T.R., Yucra H.R., Durand A.E., Hamaker B.R. (2021). Heavy metal contamination and health risk assessment in grains and grain-based processed food in Arequipa region of Peru. Chemosphere.

[112] Zeng X., Xu X., Boezen H.M., Huo X. (2016). Children with health impairments by heavy metals in an e-waste recycling area. Chemosphere.

[113] Lawal K.K., Ekeleme I.K., Onuigbo C.M., Ikpeazu V.O., Obiekezie S.O. (2021). A review on the public health implications of heavy metals. World J. Adv. Res. Rev..

[114] Zheng S., Wang Q., Yuan Y., Sun W. (2020). Human health risk assessment of heavy metals in soil and food crops in the Pearl River Delta urban agglomeration of China. Food Chem..

[115] Charkiewicz A.E., Omeljaniuk W.J., Nowak K., Garley M., Nikliński J. (2023). Cadmium toxicity and health effects—a brief summary. Molecules.

[116] Reyes-Hinojosa D., Lozada-Pérez C.A., Zamudio Cuevas Y., López-Reyes A., Martínez-Nava G., Fernández-Torres J., Olivos-Meza A., Landa-Solis C., Gutiérrez-Ruiz M.C., Rojas del Castillo E., Martínez-Flores K. (2019). Toxicity of cadmium in musculoskeletal diseases. Environ. Toxicol. Pharmacol..

[117] Wang Z., Sun Y., Yao W., Ba Q., Wang H. (2021). Effects of Cadmium Exposure on the Immune System and Immunoregulation. Front. Immunol..

[118] Collin M.S., Venkatraman S.K., Vijayakumar N., Kanimozhi V., Arbaaz S.M., Stacey R.G.S., Anusha J., Choudhary R., Lvov V., Tovar G.I. (2022). Bioaccumulation of lead (Pb) and its effects on human: A review. J. Hazard. Mater. Adv..

[119] Kosnett M.J., Wedeen R.P., Rothenberg S.J., Hipkins K.L., Materna B.L., Schwartz B.S., Hu H., Woolf A. (2007). Recommendations for Medical Management of Adult Lead Exposure. Environ. Health Perspect..

[120] Carocci A., Rovito N., Sinicropi M.S., Genchi G. (2013). Mercury toxicity and neurodegenerative effects. Rev. Environ. Contam. Toxicol..

[121] Yorifuji T., Yamamura Y., Nagano I., Kado Y., Shigeoka S., Fujino T. (2025). Prenatal methylmercury poisoning and neurocognitive impairment in Minamata. Sci. Total Environ..

[122] Li P., Du B., Chan H.M., Feng X. (2015). Human inorganic mercury exposure, renal effects and possible pathways in Wanshan mercury mining area, China. Environ. Res..

[123] Rahaman M.S., Rahman M.M., Mise N., Sikder M.T., Ichihara G., Uddin M.K., Kurasaki M., Ichihara S. (2021). Environmental arsenic exposure and its contribution to human diseases, toxicity mechanism and management. Environ. Pollut..

[124] Ren X., McHale C.M., Skibola C.F., Smith A.H., Smith M.T., Zhang L. (2011). An Emerging Role for Epigenetic Dysregulation in Arsenic Toxicity and Carcinogenesis. Environ. Health Perspect..

[125] Gikas P., Romanos P. (2006). Effects of tri-valent (Cr (III)) and hexa-valent (Cr (VI)) chromium on the growth of activated sludge. J. Hazard Mater..

[126] Wakeel A., Xu M., Gan Y. (2020). Chromium-Induced Reactive Oxygen Species Accumulation by Altering the Enzymatic Antioxidant System and Associated Cytotoxic, Genotoxic, Ultrastructural, and Photosynthetic Changes in Plants. Int. J. Mol. Sci..

[127] Gaetke L.M., Chow-Johnson H.S., Chow C.K. (2014). Copper: toxicological relevance and mechanisms. Arch. Toxicol..

[128] Maywald M., Rink L. (2022). Zinc in human health and infectious diseases. Biomolecules.

[129] Jeng S.-S., Chen Y.-H. (2022). Association of Zinc with Anemia. Nutrients.

[130] Jia X., Fu T., Hu B., Shi Z., Zhou L., Zhu Y. (2020). Identification of the potential risk areas for soil heavy metal pollution based on the source-sink theory. J. Hazard Mater..

[131] Cheng H., Hu Y. (2010). Lead (Pb) isotopic fingerprinting and its applications in lead pollution studies in China: A review. Environ. Pollut..

[132] Zhang X., Xu H., Tang J., Yang J., Guo Z., Xiao Y., Ge Y., Liu T., Hu Q., Ao H., Shi W. (2024). Cadmium absorption and translocation in rice plants are influenced by lower air temperatures during grain filling stage. Sci. Total Environ..

[133] Chen C., Xun P., Tsinovoi C., McClure L.A., Brockman J., MacDonald L., Cushman M., Cai J., Kamendulis L., Mackey J., He K. (2018). Urinary cadmium concentration and the risk of ischemic stroke. Neurology.

[134] Heidari S., Mostafaei S., Razazian N., Rajati M., Saeedi A., Rajati F. (2022). The effect of lead exposure on IQ test scores in children under 12 years: a systematic review and meta-analysis of case-control studies. Syst. Rev..

[135] Chen Y., Zhao J., Chen X., Zheng L. (2023). Analysis of the source apportionment and pathways of heavy metals in soil in a coal mining area based on machine learning and an APCS-MLR model. Minerals.

[136] Zhang H., Duan Q., Sun L., Lee J., Wu W., Zhou C., Zhang H., Guo Z., Zhang X., Tang X. (2025). A multi-component heavy metal detection method using UV-Vis superimposed spectrum and deep learning. J. Hazard Mater..

[137] Lu X., Sun L., Zhang Y., Du J., Wang G., Huang X., Li X., Wang X. (2024). Predicting Cd accumulation in crops and identifying nonlinear effects of multiple environmental factors based on machine learning models. Sci. Total Environ..

[138] Wang H., Xiao S., Shen R., Cheng Q., Zhang S. (2025). Rapid detection of soil heavy metal pollution using hyperspectral data and multiscale spatial network. Environmental Technology & Innovation.

[139] Wen Y., Wang X., Liu M., Wu L., Chen G. (2024). Identification of heavy metal stress in rice using spatial clustering based on time series of crop spectral information. Environ. Earth Sci..

[140] Polders C., Van den Abeele L., Derden A., Huybrechts D. (2012). Methodology for determining emission levels associated with the best available techniques for industrial waste water. J. Clean. Prod..

[141] Battaglia-Brunet F., Le Guédard M., Faure O., Charron M., Hube D., Devau N., Joulian C., Thouin H., Hellal J. (2021). Influence of agricultural amendments on arsenic biogeochemistry and phytotoxicity in a soil polluted by the destruction of arsenic-containing shells. J. Hazard Mater..

[142] Abhilash P.C., Tripathi V., Edrisi S.A., Dubey R.K., Bakshi M., Dubey P.K., Singh H.B., Ebbs S.D. (2016). Sustainability of crop production from polluted lands. Energy Ecol. Environ..

[143] Kong L., Guo Z., Peng C., Xiao X., He Y. (2021). Factors influencing the effectiveness of liming on cadmium reduction in rice: A meta-analysis and decision tree analysis. Sci. Total Environ..

[144] Qiu Y., Zhang Q., Gao B., Li M., Fan Z., Sang W., Hao H., Wei X. (2020). Removal mechanisms of Cr (VI) and Cr (III) by biochar supported nanosized zero-valent iron: Synergy of adsorption, reduction and transformation. Environ. Pollut..

[145] Singh S., Kapoor D., Khasnabis S., Singh J., Ramamurthy P.C. (2021). Mechanism and kinetics of adsorption and removal of heavy metals from wastewater using nanomaterials. Environ. Chem. Lett..

[146] Li H., Dong X., da Silva E.B., de Oliveira L.M., Chen Y., Ma L.Q. (2017). Mechanisms of metal sorption by biochars: Biochar characteristics and modifications. Chemosphere.

[147] Akhtar M.N., Shaikh A.J., Khan A., Awais H., Bakar E.A., Othman A.R. (2021). Smart Sensing with Edge Computing in Precision Agriculture for Soil Assessment and Heavy Metal Monitoring: A Review. Agriculture.

[148] Abhilash P.C., Powell J.R., Singh H.B., Singh B.K. (2012). Plant–microbe interactions: novel applications for exploitation in multipurpose remediation technologies. Trends Biotechnol..

[149] Tang J., Qiu Z., Tang H., Wang H., Sima W., Liang C., Liao Y., Li Z., Wan S., Dong J. (2021). Coupled with EDDS and approaching anode technique enhanced electrokinetic remediation removal heavy metal from sludge. Environ. Pollut..

[150] Brown S.L., Henry C.L., Chaney R., Compton H., DeVolder P.S. (2003). Using municipal biosolids in combination with other residuals to restore metal-contaminated mining areas. Plant Soil.

[151] Rodriguez-Valdés E., Blanco J., Alba R., Forján R., Baragaño D., Gallego J.L.R. (2025).

[152] Sprocati A.R., Alisi C., Pinto V., Montereali M.R., Marconi P., Tasso F., Turnau K., De Giudici G., Goralska K., Bevilacqua M. (2014). Assessment of the applicability of a “toolbox” designed for microbially assisted phytoremediation: the case study at Ingurtosu mining site (Italy). Environ. Sci. Pollut. Res. Int..

[153] Cobbina S.J., Chen Y., Zhou Z., Wu X., Zhao T., Zhang Z., Feng W., Wang W., Li Q., Wu X., Yang L. (2015). Toxicity assessment due to sub-chronic exposure to individual and mixtures of four toxic heavy metals. J. Hazard Mater..

[154] Monrad M., Ersbøll A.K., Sørensen M., Baastrup R., Hansen B., Gammelmark A., Tjønneland A., Overvad K., Raaschou-Nielsen O. (2017). Low-level arsenic in drinking water and risk of incident myocardial infarction: A cohort study. Environ. Res..

[155] Bjørklund G., Mutter J., Aaseth J. (2017). Metal chelators and neurotoxicity: lead, mercury, and arsenic. Arch. Toxicol..

[156] Weiskirchen R. (2025). Comprehensive Pharmacological Management of Wilson’s Disease: Mechanisms, Clinical Strategies, and Emerging Therapeutic Innovations. Science.

[157] Zhai Q., Narbad A., Chen W. (2015). Dietary strategies for the treatment of cadmium and lead toxicity. Nutrients.

[158] Gamble M.V., Liu X., Ahsan H., Pilsner J.R., Ilievski V., Slavkovich V., Parvez F., Chen Y., Levy D., Factor-Litvak P., Graziano J.H. (2006). Folate and arsenic metabolism: a double-blind, placebo-controlled folic acid–supplementation trial in Bangladesh2. Am. J. Clin. Nutr..

[159] Ho H.-J., Shirakawa H. (2022). Oxidative Stress and Mitochondrial Dysfunction in Chronic Kidney Disease. Cells.

