# Supplementary material for: The hazards of metal exposure mediated by crops to human health from perspective of environmental health and control strategies
Source: iScience. 2026 Jun 8;29(6):116147. doi: 10.1016/j.isci.2026.116147 (PMC13264138; doi:10.1016/j.isci.2026.116147)
Supplement: Document S1. Tables S1–S13 [file mmc1.pdf]

iScience, Volume *29*

Supplemental information

The hazards of metal exposure mediated by crops to human health from perspective of environmental health and control strategies

Huanhuan Yang, Fulan Zhang, Jiacun Zhou, Jiyao Zhu, Dayong Cui, Xu Zhang, and Zhibin Zhang

Supplemental data

Table S1 Overview of Mercury (Hg) Environmental Behavior and Health Risks

Indicator	Specific Content
**Main Sources**	Chemical plants, coal-fired power plants, mercury-containing pesticides, gold mining, instrument manufacturing
**Soil Concentration Range**	Background: 0.02–0.08 mg/kg; Polluted areas: Up to 10 mg/kg
**Typical Accumulating Crops**	Rice, eggplant, tomato, corn
**Human Target Organs**	Central nervous system, kidneys, liver
**Main Health Hazards**	Neurological symptoms (tremor, sensory impairment, speech disorder), renal damage, fetal malformations
**Classic Case**	Japan's "Minamata disease": Methylmercury pollution caused thousands of severe cases and long-term health damage in tens of thousands.
**International Convention & Standards**	Minamata Convention effective 2017; Drinking water Hg limit 0.001 mg/L [WHO standard]

Note: The table and all related contents were compiled by the author.

Table S2 Overview of Arsenic (As) Environmental Behavior and Health Risks

Indicator	Specific Content
**Main Sources**	Geological background, mining wastewater, arsenic pesticides (lead arsenate), smelting emissions, coal combustion, phosphate fertilizer production, etc.
**Soil Concentration Range**	Background: 5–15 mg/kg; Polluted areas: Can exceed 100 mg/kg
**Typical Accumulating Crops**	Rice, carrot, radish, potato, and other root vegetables
**Human Target Organs**	Skin, liver, lungs, cardiovascular system, nervous system
**Main Health Hazards**	Chronic arsenicism (melanosis, skin keratosis); Liver, lung, bladder cancer, etc.
**Typical Case**	Bangladesh groundwater arsenic contamination incident: Over 40 million people affected.
**Environmental & Food Limits**	Drinking water As limit 0.01 mg/L [WHO standard]; Rice As limit 0.2 mg/kg [GB2762-2022]

Note: The table and all related contents were compiled by the author.

Table S3 Overview of Chromium (Cr) Environmental Behavior and Health Risks

Indicator	Specific Content
**Main Sources**	Tanneries, electroplating industry, dye manufacturing, smelting wastewater, chromium slag dump sites
**Soil Concentration Range**	Background: 60–90 mg/kg; Polluted areas: 400–1500 mg/kg
**Typical Accumulating Crops**	Spinach, carrot, Chinese cabbage, potato
**Human Target Organs**	Respiratory system, kidneys, liver, skin
**Main Health Hazards**	Lung cancer, skin cancer, nasopharyngeal carcinoma, respiratory diseases, renal damage
**Typical Case**	Soil Cr content reached 1500 mg/kg at a chromium slag site in Guizhou; Chromium slag groundwater pollution incident in Karnataka, India.
**Environmental & Food Limits**	Soil Cr limit ≤150 mg/kg; Total Cr in drinking water ≤0.05 mg/L [GB5749-2022]

Note: The table and all related contents were compiled by the author.

Table S4 Environmental Behavior and Health Risk Overview of Other Heavy Metals

Element	Function/Risk Profile	Main Sources	Soil Concentration Range (mg/kg)	Accumulating Crops	Health Hazards	Environmental Standard Reference
**Cu**	Essential element; excess→liver/kidney damage	Smelting, pesticides, sludge, mining	Background: 20–50; Polluted: >200	Leafy vegetables, root crops	Liver damage, gastrointestinal disorders	Soil Cu limit ≤50 [GB5749-2022] EU**:** EC 1881/2006 (cereal Cu limit 10 mg/kg) EU**:** EC 1881/2006 (cereal Zn limit 50 mg/kg)
**Zn**	Essential element; excess→immune suppression	Fertilizers, feed, industrial emissions	Background: 60–100; Polluted: >300	Corn, rice, potato	GI irritation, immune suppression, anemia	Soil Zn limit ≤200 [GB5749-2022]
**Ni**	Industrial metal; strong sensitizer, carcinogen	Smelting, alloys, nickel ore	Background: 10–30; Polluted: >200	Root crops	Nasal cancer, lung cancer, dermatitis	Soil Ni limit ≤40 [GB5749-2022]

Element	Function/Risk Profile	Main Sources	Soil Concentration Range (mg/kg)	Accumulating Crops	Health Hazards	Environmental Standard Reference
**Co**	Trace element; excess→cardiovascular risk	Battery manufacturing, hard alloys,mining	Background: 5–10; Polluted: >40	Root crops	Myocardial damage, thyroid abnormalities	Soil Co limit ≤20 [GB5749-2022]

Note: The table and all related contents were compiled by the author.

Table S5 Heavy Metal Input Characteristics and Risk Management Measures for Agricultural Pollution Sources

Agricultural Pollution Input Pathway	Typical Metals	Risk Parameters/Limits	High-Risk Crops	Risk Characteristics	Management Suggestions
**Phosphate Fertilizer (Natural Cd)**	Cd	EU product limit: ≤60→20 mg/kg P_2O_5	Rice, Leafy veg	High Cd availability in acidic soil	Use low-Cd P fertilizer, lime application, organic amendments
**Sludge/Biosolids**	Cu, Zn, Pb, Cd, Hg	Cd: 20–40; Pb: 750–1200 mg/kg DM	Leafy, Root veg	OM complexes reduce short-term availability; Long-term accumulation	Test per directive; Control application rate; Link with soil pH
**Urban Wastewater/Reclaimed Water**	Multiple metals	Global irrigated area 29.3 Mha, 885M exposed	Rice, Leafy veg	Colloidal input; High frequency irrigation accumulates	WHO multiple barrier approach (source→field→washing)
**Livestock Manure**	Cu, Zn	Feed Cu/Zn addition limits	Corn, Wheat, Veg	Cu/Zn concentration significantly higher after years of use	Feed control; Regular soil testing
**Historical Pesticide Legacy**	Pb, As	Soil risk screening value	Root crops, Gardens	Pb/As co-occurrence fingerprint, long-term risk	Amendment/soil replacement/raised bed planting

Note: The table and all related contents were compiled by the author.

Table S6 Heavy Metal Input Characteristics and Risk Management Measures for Domestic Pollution Sources

Domestic Pollution Input Pathway	Typical Metals	Risk Parameters/ Range	High-Risk Crops	Risk Characteristics	Management Suggestions
**Greywater/Domestic Sewage Irrigation**	Cu, Zn, Pb, Cd	Global downstream farmland 29.3 Mha, 885M exposed	Leafy veg, Rice	High metal concentration in water, frequent application; Long-term accumulation, easy enrichment in leaves/grains	Switch to drip/furrow irrigation; Construct small wetlands; Post-harvest washing/peeling
**Homemade Compost (mixed waste)**	Pb, Cd	Some compost Pb>100; Cd>3	Garden vegetables	Pollution from mixed batteries, pigments, metal scraps	Publicize waste sorting; Compost metal spot checks; Encourage certified organic fertilizer
**Informal Recycling/Dismantling Sites**	Pb, Cu, Cd, Hg, Sb	Dismantling area soil Pb>500, Cu>400	Rice paddies, Gardens	Acid washing and incineration produce settled dust; High exposure risk via food chain	Industrial park management; Soil/sediment cleanup; Ban planting or rotate low-accumulation crops in risk areas
**Historical Orchards/Lead Arsenate Legacy**	Pb, As	Soil Pb>300; As>50	Root crops, Gardens	Pb/As co-occurrence fingerprint, long-term pollution, low mobility	Soil amendment/replacement; Pre-development risk survey; Plant non-edible/ornamental crops

Note: The table and all related contents were compiled by the author.

Table S7 Key Environmental Factors Influencing Rhizosphere Heavy Metal Uptake

Factor	Impact on Heavy Metal Activity and Uptake
**Soil pH**	pH decrease → Cd, Pb, Ni, Zn activity increase; Alkalization can immobilize metals
**Eh (Redox Potential)**	Reducing conditions release As, Fe, Mn, promoting arsenic activity
**Organic Matter**	Provides complexation sites, can immobilize or increase mobility
**Fe/Mn Oxides**	Adsorb and co-precipitate heavy metals, important immobilizers
**Rhizosphere**	Phosphate-solubilizing, iron-reducing bacteria regulate metal speciation;

Factor	Impact on Heavy Metal Activity and Uptake
**Microbes**	Mycorrhizal fungi enhance resistance
**Root Exudates**	Organic acids, amino acids, flavonoids promote or regulate metal uptake

Note: The table and all related contents were compiled by the author.

Table S8 Major Transmembrane Transporter Proteins for Heavy Metals in Plants System

Metal	Main Transporter/Protein	Functional Characteristics
**Cd**	IRT1, ZIP, HMA2/HMA3	Transmembrane uptake and xylem transport, key pathways for Cd shoot translocation
**Pb**	Ca channels, HMA, ABC	Mostly fixed in roots, weak translocation to grains
**As**	Lsi1/Lsi2, NIP	Arsenic enters rice grains as As(III) via silicon channels
**Cr**	NRAMP, Anion channels	Cr(VI) enters via anion channels, more toxic
**Zn/Cu**	ZIP, YSL, COPT	Essential element transport systems, prone to overload at high concentrations
**Ni**	NRAMP, ZIP	Significant leaf accumulation, high concentrations impair chlorophyll synthesis

Note: The table and all related contents were compiled by the author.

Table S9 Impact of Heavy Metal Stress on Growth and Yield of Major Crops

Crop	Heavy Metal	Stress Concentration Range (mg/kg soil)	Main Morphological Symptoms	Yield Change
**Rice**	As, Cd	As>10; Cd>2	Seedling stunting, leaf tip yellowing, poor grain filling	Yield loss 15–30%
**Wheat**	Cd, Pb	Cd>3; Pb>100	Short thick roots, leaf chlorosis, low grain plumpness	Yield loss 20–40%
**Corn**	Cr, Ni	Cr>200; Ni>50	Leaf margin yellowing/curling	Yield loss 15–25%
**Spinach**	Pb, Hg	Pb>300; Hg>5	Leaf spots, chlorosis	Marketability significantly reduced
**Potato, Carrot**	Pb, Cr	Pb>300; Cr>150	Tuber skin accumulation, malformation	Quality decline

Note: The table and all related contents were compiled by the author.

Table S10 Characteristics of Plant Antioxidant System Response under Heavy Metal Stress

Heavy Metal	ROS Accumulation Feature	Antioxidant Enzyme Trend	Non-enzymatic Antioxidant Change	Physiological Significance
**Cd**	Rapid ROS accumulation	SOD, CAT early rise, long-term fall	GSH, PCs content increase	Alleviate Cd toxicity via complexation/sequestration

Heavy Metal	ROS Accumulation Feature	Antioxidant Enzyme Trend	Non-enzymatic Antioxidant Change	Physiological Significance
**Pb**	H_2O_2 accumulation significant	POD, APX enhance	GSH decreases	Pb mainly fixed in cell walls, ROS damage leaves
**As**	As(V) reduction produces ROS	GR, APX significantly increase	PCs, sulfur metabolism enhance	Significant GSH consumption, affects S metabolism
**Cr(VI)**	High oxidative pressure	SOD, CAT inhibited	MDA content increases	Strong Cr oxidation causes severe lipid peroxidation

Note: The table and all related contents were compiled by the author.

Table S11 Signaling Molecules and Regulatory Factors Associated with Heavy Metal Stress

Signaling Pathway/Factor	Target	Functional Description
**HMA2/HMA3**	Cd, Zn	Regulation of root-to-shoot transport, vacuolar sequestration
**NRAMP Family**	Mn, Fe, Cd	Transmembrane uptake and intracellular migration of metals
**Lsi1/Lsi2 Channels**	As(III)	Determines arsenic uptake capacity in rice
**ABC Transporters**	Various metals	Transmembrane pumping of metal-chelate complexes to vacuoles or extracellular space
**WRKY, MYB, NAC TFs**	Metal chelation & antioxidation	Activate defense genes, enhance resistance
**miR398, miR395**	SOD, Sulfur metabolism	Respond to Cd, As stress, regulate antioxidation and chelator metabolism

Note: The table and all related contents were compiled by the author.

Table S12 Overview of Toxic Target Organs and Health Hazards of Major Heavy Metals

Heavy Metal	Main Target Organs	Typical Health Hazards	Typical Case/Data
**Cd**	Kidneys, Bones	Renal damage, osteoporosis, anemia	Itai-itai disease; Fracture rate >65%[160]
**Pb**	Nervous system, Hematopoietic system, Kidneys	Child intellectual decline, anemia, hypertension	800M children globally with blood Pb ≥50 μg/L[161]
**Hg**	Nervous system, Kidneys	Minamata disease: sensory disturbance, fetal malformations	~15 million people threatened globally[162]
**As**	Skin, Cardiovascular, Respiratory systems	Skin lesions, multiple cancer risks	40M residents in South Asia drink high-As water[163]
**Cr**	Respiratory system, Liver, Skin	Nasal septum perforation, lung cancer	Electroplating worker lung cancer rate 3 to 5 times higher[164]

Heavy Metal	Main Target Organs	Typical Health Hazards	Typical Case/Data
**Cu**	Liver, Nervous system	Wilson's disease, cirrhosis, gastroenteritis	Wilson's patient cirrhosis rate >50%[165]
**Zn**	Gastrointestinal, Hematopoietic systems	GI discomfort, anemia, immune dysfunction	High-Zn water residents anemia prevalence increased by approximately 20%[166]

Note: The table and all related contents were compiled by the author.

Table S13 Full-Chain Monitoring Indicators and Risk Thresholds for Heavy Metal Pollution

Stage	Monitoring Indicator	Typical Threshold	Risk Determination	Remarks
**Source (Soil)**	Soil Cd	> 0.3 mg/kg (Farmland risk screening value)	Risk of agricultural product 超标	'Soil Environmental Quality Risk Control Standard for Farmland (GB15618-2018)'
	Soil Pb	> 80 mg/kg	Health risk exists	Restrict edible crop planting in high-risk areas
	Soil As	> 25 mg/kg	Potential carcinogenic risk	Delineate control areas
**Process (Crops)**	Rice grain Cd	> 0.2 mg/kg	Not suitable for consumption	'National Food Safety Standard for Contaminants (GB2762-2022)'
	Vegetable Pb content	> 0.1 mg/kg	Consumption alert	Focus on leafy vegetables
	Root crop Cd accumulation factor	> 0.5	High risk	Adjust planting structure
**Population (Health)**	Child blood lead	> 50 µg/L	Initiate intervention	Affects intelligence & neurodevelopment
	Adult urinary Cd	> 5 µg/g creatinine	Increased renal damage risk	Implement health check-ups & follow-up
	Hair Hg content	> 2 µg/g	Long-term exposure risk	Suitable for coastal/mining populations
**Risk (Dietary Exposure)**	Adult daily rice Cd intake	> 0.8 µg/kg body weight	Exceeds Tolerable Weekly Intake (TWI)	EFSA (2011) health risk reference value

Note: The table and all related contents were compiled by the author.
